# The pan-AMPK activator MK-8722 outperforms the AMPK activators SC4 and A-769662 in human endothelial cells

**DOI:** 10.1042/BSR20250408

**Published:** 2026-09-22

**Authors:** Heena Doshi, Katrin Spengler, Kanstantsin Siniuk, Marco Groth, Estelle Heyne, Michael Schwarzer, Amod Godbole, Yi Sing Gee, Jonathan Baell, Jonathan S. Oakhill, Andreas Henke, Regine Heller

**Affiliations:** 1 Institute for Molecular Cell Biology, Center for Molecular Biomedicine, Jena University Hospital, 07745 Jena, Germany; 2 Leibniz Institute on Aging- Fritz Lipmann Institute (FLI), 07745 Jena, Germany; 3 Department of Cardiothoracic Surgery, Jena University Hospital, 07747 Jena, Germany; 4 Medicinal Chemistry, Monash Institute of Pharmaceutical Sciences, Monash University, Parkville 3052, Australia; 5 School of Pharmaceutical Sciences, Nanjing Tech University, Nanjing 211816, People’s Republic of China; 6 Metabolic Signaling Laboratory, St. Vincent’s Institute of Medical Research, Fitzroy 3065, Australia; 7 Faculty of Health Sciences, Australian Catholic University, Melbourne 3065, Australia; 8 Section of Experimental Virology, Institute of Medical Microbiology, Jena University Hospital, 07745 Jena, Germany

**Keywords:** A-769662, AMPK, AMPK activators, Endothelial cells, MK-8722, SC4

## Abstract

AMP-activated protein kinase (AMPK), a heterotrimeric serine/threonine protein kinase consisting of the catalytic α-subunit and regulatory β- and γ-subunits, regulates endothelial homeostasis. Consequently, it has emerged as a target for pharmacological activators in the treatment of endothelial dysfunction. However, the efficiency of these agonists may differ depending on the expression of different AMPK subunit isoforms in different cells and tissues, as these isoforms may form AMPK complexes with distinct activation profiles. The present study compares the effects of three direct AMPK activators in endothelial cells: MK-8722, a pan-AMPK activator; SC4, an intermediate activator with preference for AMPKα2; and A-769662, a β1-specific compound. We demonstrate that MK-8722 (0.1 to 10 μM) induces a robust and sustained, short- and long-term activation of AMPK, as evidenced by the phosphorylation of acetyl-CoA carboxylase and the inhibition of the mechanistic target of rapamycin complex 1 pathway. On an equimolar basis, MK-8722 was significantly more potent than SC4 and A-769662. This was associated with a significant antiviral effect of MK-8722 against herpes simplex virus type 1, whereas SC4 and A-769662 had no effect. At 10 μM, MK-8722 led to energy depletion and increased formation of mitochondrial and cytosolic reactive oxygen species due to inhibition of mitochondrial complex I. Under these conditions, liver kinase B1-mediated AMPK activation was observed but was not functionally relevant. We propose that the strong activation of AMPK by MK-8722 is related to the presence of different AMPK heterotrimers in endothelial cells. Therefore, targeting endothelial dysfunction pharmacologically may require pan-AMPK activators.

## Introduction

Endothelial cells form a monolayer lining the inner walls of the entire vasculature. They play a vital role in maintaining vascular homeostasis by regulating processes such as barrier function, nutrient exchange, vasoreactivity, and angiogenesis. This involves responding to various physiological and pathological stimuli via different signalling pathways. Dysfunctional endothelium has been linked to diseases such as atherosclerosis, diabetes, and hypertension. Therefore, maintaining the function of endothelial cells is crucial for cardiovascular health [1–3].

The AMP-activated protein kinase (AMPK) is one of the signalling proteins responsible for maintaining endothelial homeostasis [4,5]. It is a heterotrimeric serine/threonine kinase consisting of a catalytic α-subunit as well as regulatory β- and γ-subunits [6,7]. There are two α- and two β-isoforms, as well as three γ-isoforms, which can form 12 different complexes with unique biochemical properties. AMPK senses changes in nutrient and energy levels. It is activated in response to an increase in the AMP/ATP and ADP/ATP ratios via three mechanisms: allosteric activation by AMP binding to the γ-subunit; phosphorylation of threonine 172 (T172) on the activation loop of AMPKα; and inhibition of dephosphorylation of T172 [8]. AMPK’s upstream kinases include liver kinase B1 (LKB1) in response to energy depletion and calcium (Ca^2+^)/calmodulin-dependent protein kinase kinase 2 (CaMKK2) in response to Ca^2+^-elevating agonists, including hormones and growth factors [9]. Upon activation, AMPK orchestrates a metabolic switch that promotes catabolic pathways such as fatty acid oxidation, mitochondrial biogenesis, and glucose uptake, which produce energy. It also inhibits anabolic pathways such as lipid and protein synthesis, which consume energy [6,10,11]. In endothelial cells, AMPK plays a multifaceted role in addition to its metabolic regulatory function, in part by exploiting Ca^2+^-dependent activation mechanisms. AMPK is essential for maintaining endothelial barrier function, promoting angiogenesis, and safeguarding endothelial cells against oxidative and inflammatory stress [4,5,12,13] and has antiviral effects [14–16]. Therefore, activating AMPK may prevent endothelial dysfunction. In line with this, metformin, a well-known AMPK agonist and antidiabetic drug, has been shown to exert vascular protective effects independent of its glucose-lowering properties [17,18].

Due to its metabolic effects, AMPK has emerged as a therapeutic target for metabolic and chronic diseases such as obesity, insulin resistance, and type 2 diabetes [19–23]. Consequently, a variety of pharmaceutical and nutraceutical AMPK activators are being developed and tested in preclinical and clinical studies. AMPK agonists generally act via direct and indirect mechanisms. Indirect activators increase the cellular AMP/ADP:ATP ratio, primarily by inhibiting the mitochondrial respiratory chain. Examples of such activators include metformin, resveratrol, berberine, or quercetine [24]. Direct activators lead to allosteric activation of AMPK by mimicking the action of AMP or by binding to the so-called allosteric drug and metabolite (ADaM) site of AMPK [25,26]. This is a pocket formed between the amino-terminal lobe of the kinase domain and the carbohydrate-binding module of the β-subunit [27]. For example, 5-aminoimidazole-4-carboxamide ribonucleotide (also known as ZMP), the derivative of the cell-permeable precursor AICAR, interacts with the nucleotide binding site of the γ-subunit, while salicylate, A-769662, MK-8722, and SC4 bind to the ADaM site. A-769662 is a thienopyridine drug that requires the β1-regulatory subunit and phosphorylation at its serine residue 108 for AMPK activation [27–30]. MK-8722 binds to both β-isoforms, albeit with slightly higher affinity for the β1-isoform, and can activate all 12 heterotrimeric AMPK complexes [31,32]. SC4 activates both β1- and β2-containing AMPK complexes, showing some selectivity for AMPKα2-containing complexes [33].

Although there is strong evidence to support the hypothesis that activating AMPK is beneficial in preventing and treating various chronic diseases, this strategy requires careful, targeted, and context-specific intervention, given the tissue- and isoform-specific roles of AMPK [19]. In line with this, the effectiveness of AMPK activators on target and non-target tissues, as well as their potential adverse effects, must be examined in order to identify suitable compounds for therapeutic use. Testing AMPK activators in endothelial cells in particular will identify candidates for the treatment of endothelial dysfunction. Against this background, we studied the effects of three AMPK activators, MK-8722, SC4, and A-769662, on the phosphorylation of downstream targets of AMPK (acetyl-CoA carboxylase (ACC) and regulatory-associated protein of mechanistic target of rapamycin complex 1 (mTORC1; Raptor)) and cellular energy metabolism in endothelial cells. Building on our previous study of the antiviral effects of AMPK [16], we also compared the impact of these three AMPK activators on the replication of herpes simplex virus type 1 (HSV-1) in endothelial cells. Our study revealed different AMPK activation profiles for the compounds tested, with MK-8722 demonstrating the greatest efficiency and suggesting that pan-AMPK activators may be required to target endothelial dysfunction.

## Results

### MK-8722 is the most potent AMPK activator in endothelial cells

**Figure 1:**
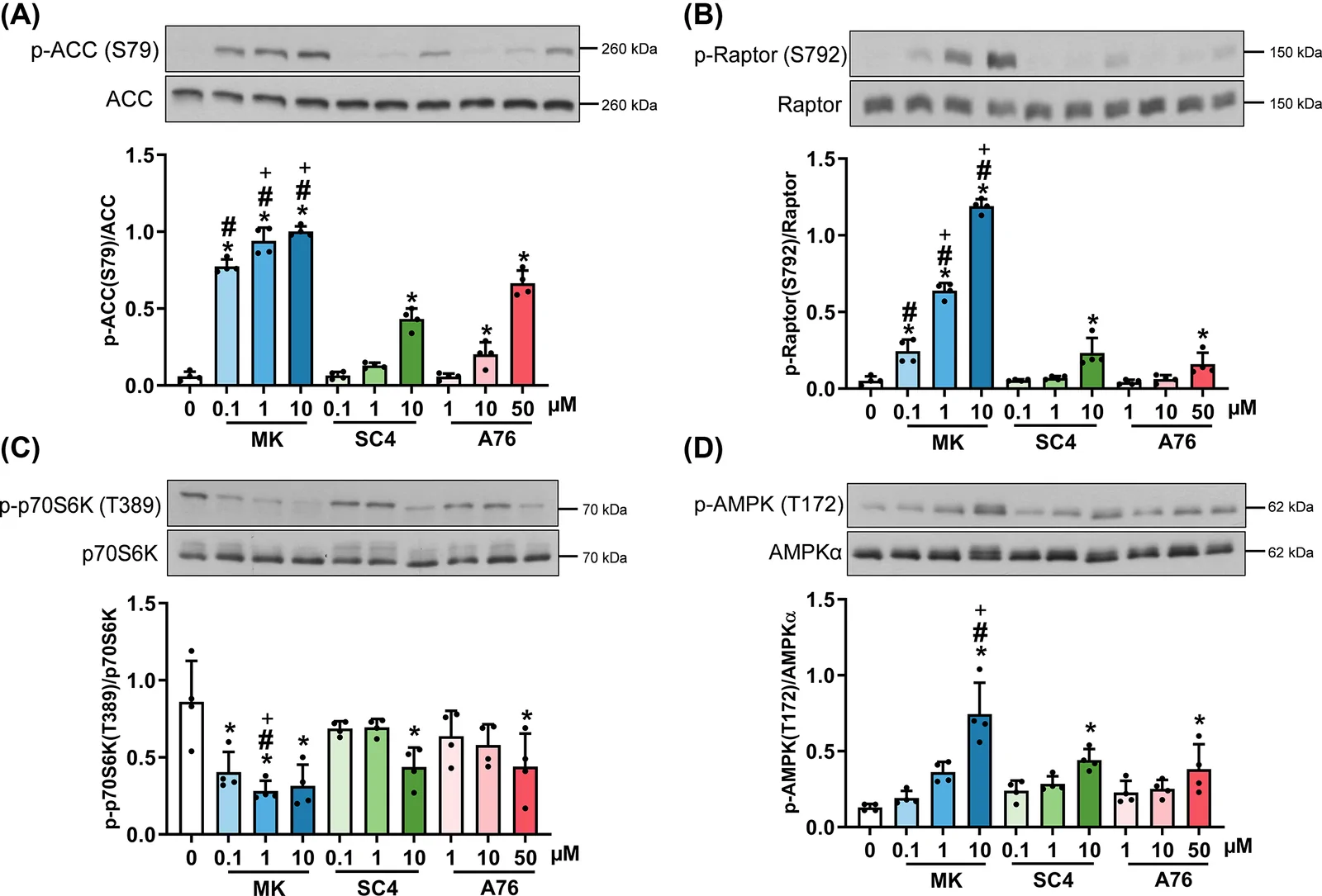
Activation of the AMPK pathway by MK-8722, SC4, and A-769662—short-term incubations HUVEC were treated with MK-8722 (MK), SC4 and A-769662 (A76) at the indicated concentrations for 15 min. Cells were lysed and subjected to Western blot analysis of ACC phosphorylated at S79 (**A**), Raptor phosphorylated at S792 (**B**), p70S6K phosphorylated at T389 (**C**), AMPK phosphorylated at T172 (**D**) and the corresponding non-phosphorylated proteins. Representative immunoblots and quantification by densitometry with normalisation of phosphorylated proteins to total proteins are shown. Mean + SD, *n* = 4. *P<0.05 versus the untreated control, ^#^*P<*0.05 versus equimolar doses of SC4, ^+^*P<*0.05 versus equimolar doses of A-769662 using one-way repeated measurement analysis of variance (ANOVA) corrected by the Holm–Šidák method.

**Figure 2:**
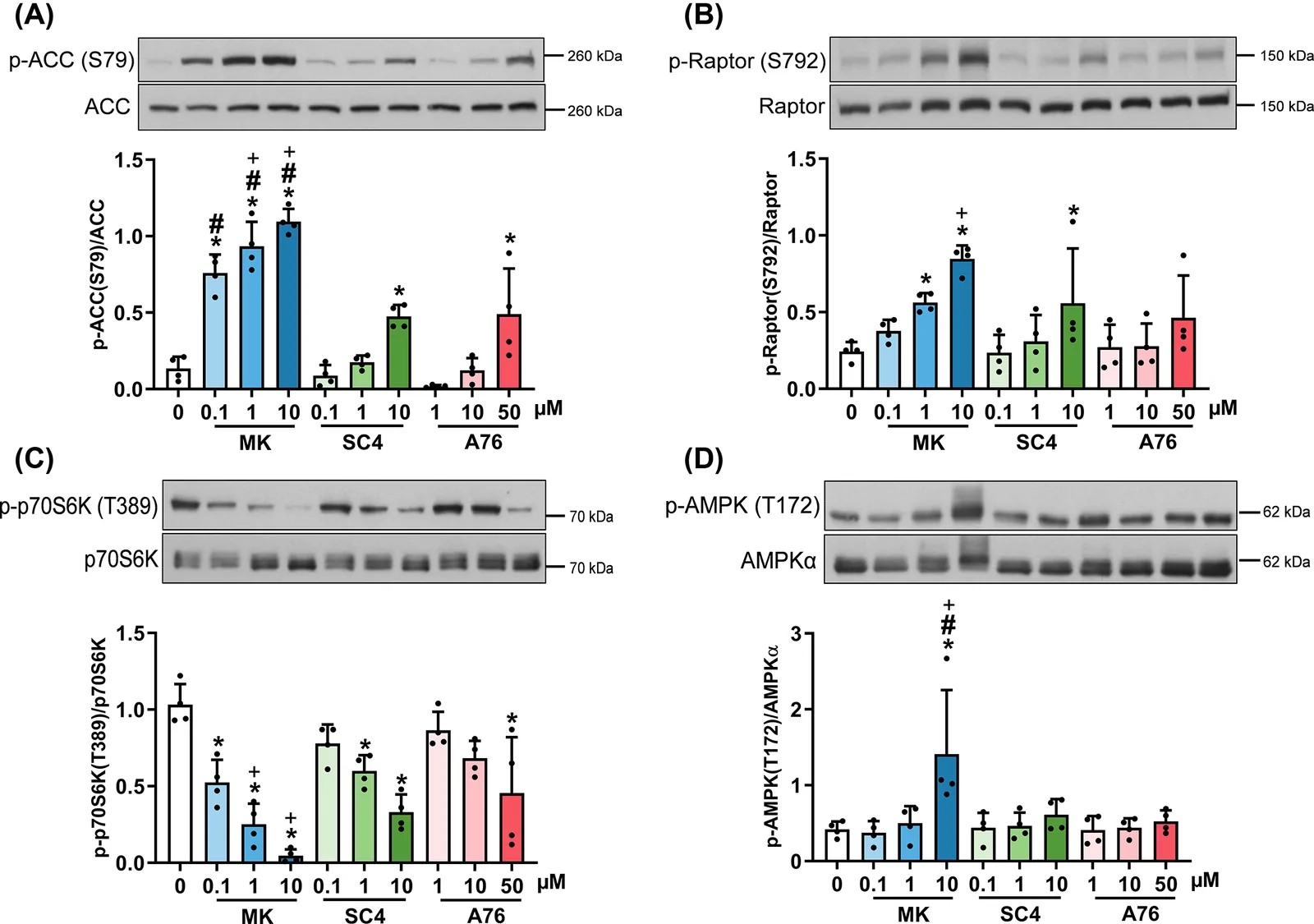
Activation of the AMPK pathway by MK-8722, SC4, and A-769662—long-term incubations HUVEC were treated with MK-8722 (MK), SC4, and A-769662 (A76) at the indicated concentrations for 24 h. Cells were lysed and subjected to Western blot analyses of ACC phosphorylated at S79 (**A**), Raptor phosphorylated at S792 (**B**), p70S6K phosphorylated at T389 (**C**), AMPK phosphorylated at T172 (**D**), and the corresponding non-phosphorylated proteins. Representative immunoblots and quantification by densitometry with normalisation of phosphorylated proteins to total proteins are presented. Mean + SD, *n* = 4. *P<0.05 versus non-treated control, ^#^*P<*0.05 versus equimolar doses of SC4, and ^+^*P<*0.05 versus equimolar doses of A-769662 using one-way repeated measurement ANOVA corrected by the Holm–Šidák method.

Firstly, we treated human umbilical vein endothelial cells (HUVEC) with the pharmacological AMPK activators MK-8722, SC4, and A-769662 at the concentrations commonly reported in the literature [29,32,33] under acute (15 min) and chronic (24 h) conditions. The compounds showed no cytotoxic effects at these concentrations (Supplementary Figure S1). Following a 15 min incubation period, MK-8722 (0.1–10 μM) was found to increase the phosphorylation of ACC (Figure 1A) and Raptor (Figure 1B) by up to 20-fold and 25-fold, respectively, compared with the untreated control, respectively, while decreasing the phosphorylation of p70S6K (Figure 1C) by 40% compared with control levels. This indicates the inhibition of ACC and the mTORC1 pathway. A strong effect was also observed following long-term treatment of endothelial cells with MK-8722 for 24 h (up to 9-fold and 3.5-fold increases compared with the untreated control in ACC (Figure 2A) and Raptor (Figure 2B) phosphorylation, respectively, and a decrease in p70S6K phosphorylation by 90% compared with control levels (Figure 2C)). In contrast, the acute and chronic treatment of endothelial cells with either SC4 or A-769662 resulted in more moderate dose-dependent increases in ACC and Raptor phosphorylation as well as smaller decreases in p70S6K phosphorylation (Figures 1 and 2). At an equimolar level (i.e., 1 or 10 μM), MK-8722 was the most potent inducer of ACC phosphorylation and mTORC1 pathway inhibition following both short- and long-term treatment. AMPK phosphorylation was only observed with the highest concentration of the compounds after 15 min and only with the highest concentration of MK-8722 (10 μM) after 24 h (Figures 1 and 2D).

### MK-8722 affects energy metabolism in endothelial cells

**Figure 3:**
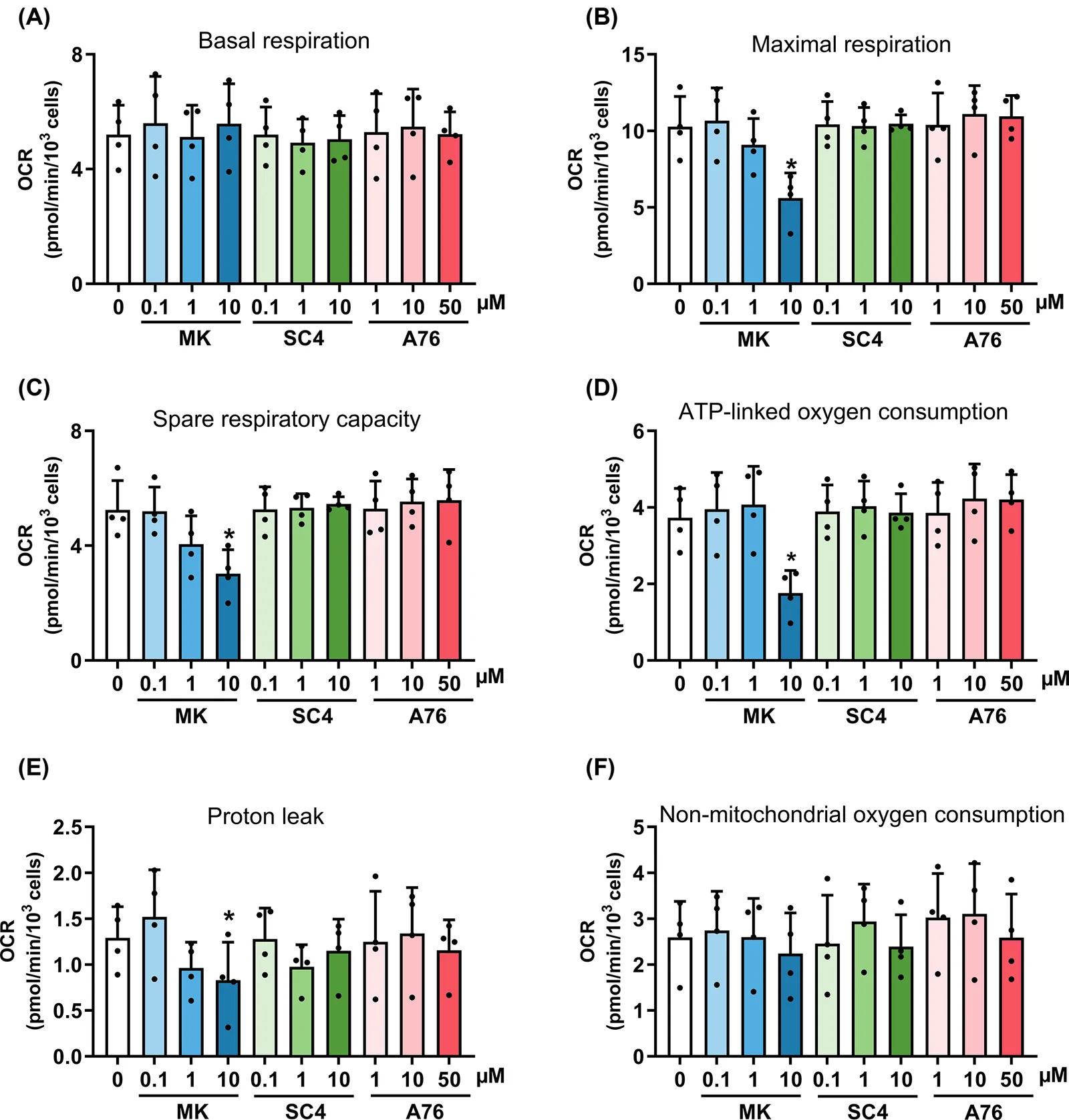
Effects of MK-8722 on mitochondrial energy metabolism in endothelial cells MK-8722 (MK), SC4, and A-769662 (A76) were added to the Seahorse assay medium at the indicated concentrations, and OCR was measured under basal conditions and following the sequential addition of compounds that modulate the respiratory chain: oligomycin (2 μM) to block the mitochondrial ATP synthase, carbonyl cyanide-4-(trifluoromethoxy) phenylhydrazone (FCCP; 2 μM) to uncouple oxidative phosphorylation, and antimycin A (2 μM) to inhibit mitochondrial respiration. Parameters of mitochondrial metabolism were then calculated from the resulting changes in OCR (basal (**A**) and maximal (**B**) respiration, spare capacity (**C**), ATP-linked oxygen consumption (**D**), proton leak (**E**), and non-mitochondrial oxygen consumption (**F**)). Mean + SD, *n* = 4. *P<0.05 versus untreated control by one-way repeated measurement ANOVA corrected by the Holm–Šidák method.

To understand whether the potent activation of the AMPK pathway by MK-8722 involves mechanisms other than allosteric ones, we examined the effect of this compound on endothelial energy metabolism using Seahorse analyses and compared it with the effects of SC4 and A-769662. MK-8722 was the only compound to affect respiratory parameters but reached significance only at 10 μM. The acute addition of MK-8722 to endothelial cells reduced mitochondrial ATP-linked oxygen consumption by 47%, maximal respiration by 55%, and spare capacity by 58%, while protecting against proton leak (Figure 3). The oxygen consumption rate (OCR) trace for the acute addition of the activators is shown in Supplementary Figure S2A. Following chronic treatment of cells with MK-8722 for 24 h, there was a reduction in basal respiration and a trend towards lower levels of mitochondrial ATP-linked oxygen consumption, maximal respiration, and spare capacity (Supplementary Figure S2B). These data suggest that at higher concentrations, MK-8722 leads to energy depletion, which in turn may potentiate allosteric AMPK activation via a mechanism involving AMP binding and LKB1-mediated phosphorylation.

### MK-8722 inhibits complex I of the mitochondrial respiratory chain

**Figure 4:**
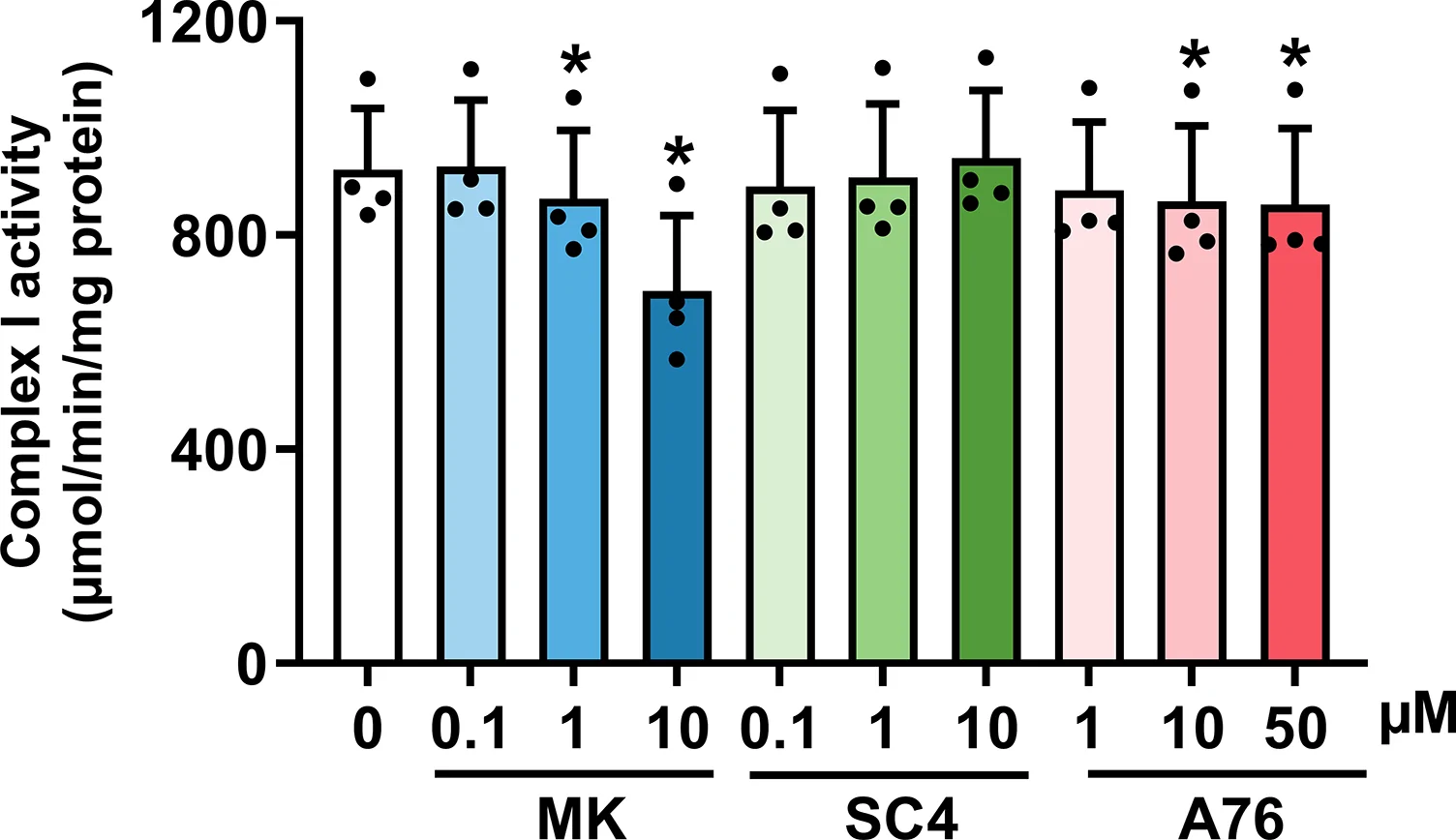
Effect of MK-8722, SC4, and A-769662 on respiratory complex activity Rat heart mitochondria were isolated and subjected to mitochondrial complex activity measurements. MK-8722 (MK), SC4, or A-769662 (A76) were added to the assay at the indicated doses. Complex I activity was normalised to the protein content of the sample. Mean + SD, *n* = 4. *P<0.05 versus untreated control by one-way repeated measurement ANOVA corrected by the Holm–Šidák method.

In the search for a mitochondrial target of MK-8722, we used isolated rat heart mitochondria to measure the effect of the tested compounds on the activity of respiratory complexes *in vitro*. As illustrated in Figure 4, MK-8722 inhibited the activity of complex I at concentrations of 1 and 10 μM, whereas it did not affect complexes II, III, or V and moderately activated complex IV (Supplementary Figure S3). Neither SC4 nor A-769662 affected the activity of the respiratory complexes, except for a slight decrease in complex I activity after treatment with 10 and 50 μM of A-769662 (Figure 4). Based on these findings, we concluded that inhibition of complex I by MK-8722 was responsible for the observed interference with mitochondrial respiration.

### MK-8722 causes moderate mitochondrial dysfunction

**Figure 5:**
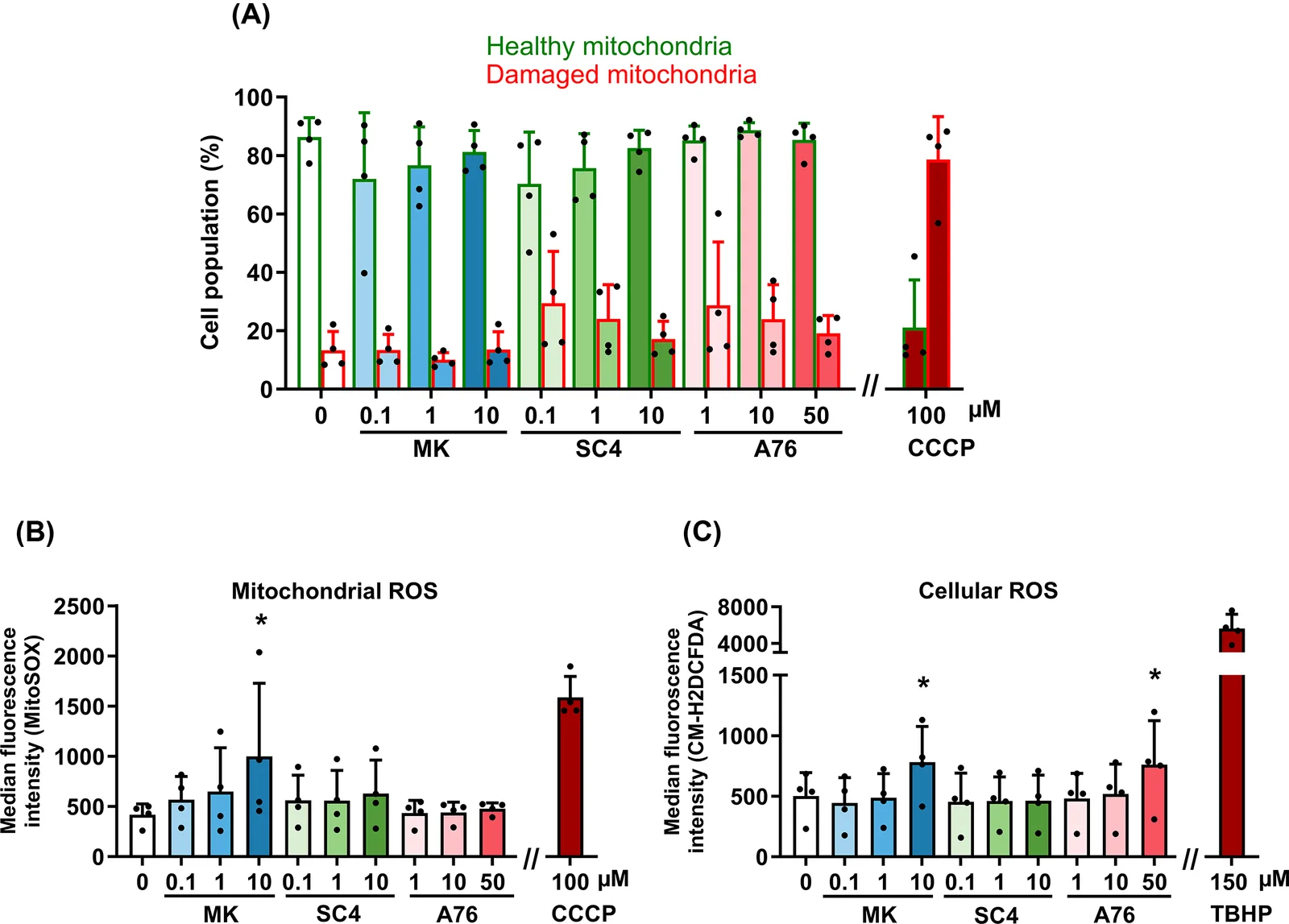
Effect of MK-8722, SC4, and A-769662 on mitochondrial stability (A–C) HUVEC were treated with MK-8722 (MK), SC4, or A-769662 (A76) at the indicated concentrations for 24 h. Mitochondrial permeability using JC-10 (**A**), mitochondrial ROS using MitoSOX (**B**), and cellular ROS using dichlorodihydrofluorescein diacetate (CM-H2DCFDA) (**C**) were measured by flow cytometry. Mean + SD, *n* = 4 *P<0.05 versus non-treated control by one-way repeated measurement ANOVA corrected by the Holm–Šidák method. Positive controls (CCCP—carbonyl cyanide m‐chlorophenylhydrazone, a mitochondrial uncoupler, 100 μM, 30 min; TBHP—tert-Butyl hydroperoxide, 150 μM, 30 min) are demonstrated in dark red bars with a scale break. They are not included in the statistics.

We examined mitochondrial permeability, reactive oxygen species (ROS) formation, and morphology to see whether inhibition of complex I activity by MK-8722 affects mitochondrial stability and morphology. Mitochondrial permeability as measured using the cationic and lipophilic mitochondrial membrane potential probe JC-10 was not altered in response to MK-8722, SC4, or A-769662 (Figure 5A). However, the mitochondrial and cytoplasmic ROS levels were increased by 10 μM MK-8722, indicating a mild mitochondrial dysfunction under these conditions (Figure 5B,C, respectively). Mitochondrial morphology was not altered as qualitatively assessed by immunofluorescence staining of the inner (ATP synthase beta subunit (ATPB)) and the outer (translocase of outer mitochondrial membrane 20 (TOMM20)) mitochondrial membranes (Supplementary Figure S4). SC4 did not induce ROS formation, and A-769662 led to a moderate increase in cytoplasmic ROS when applied at 50 μM.

### MK-8722-mediated energy depletion plays a minor role in AMPK activation

**Figure 6:**
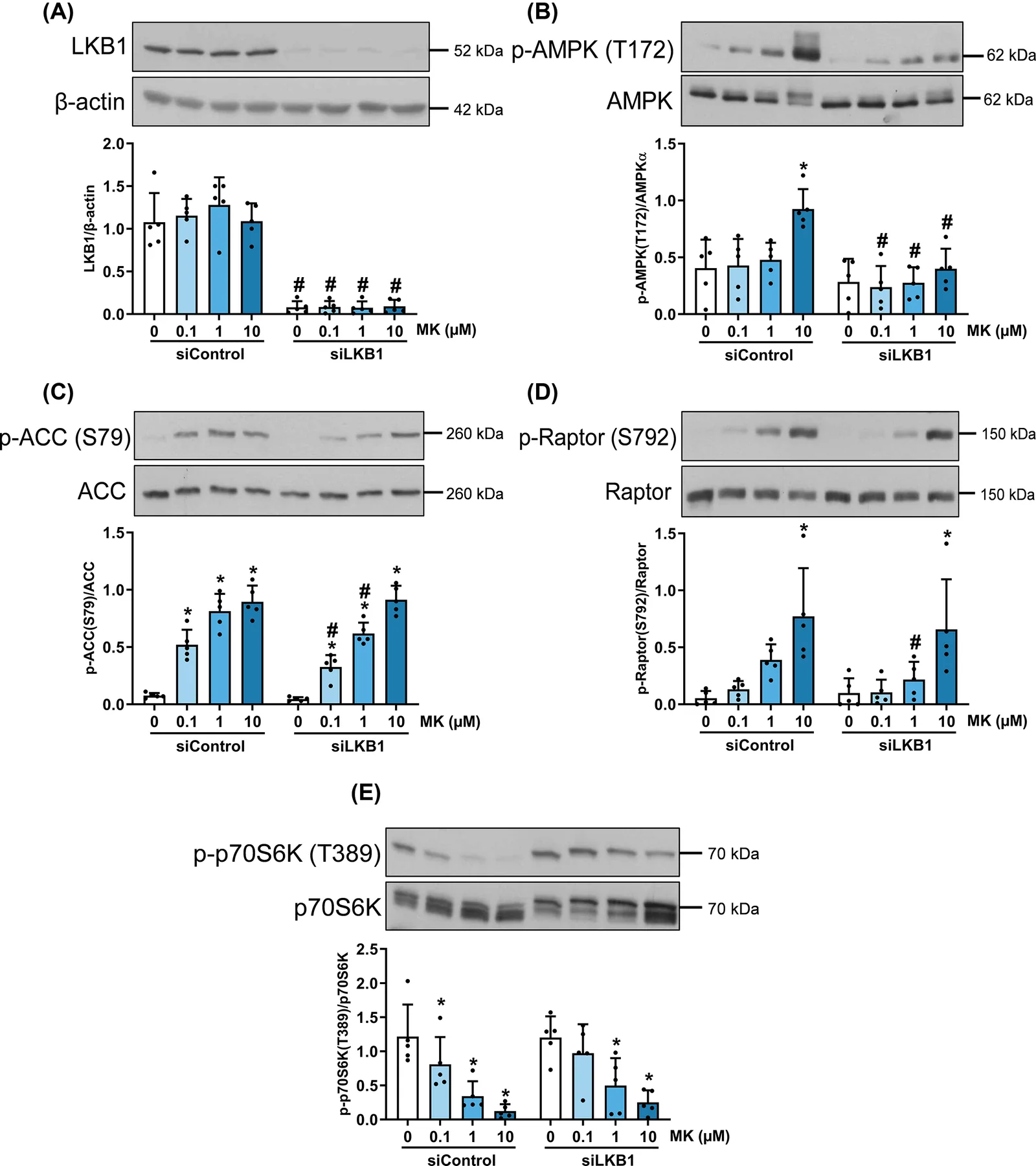
MK-8722-induced activation of the AMPK pathway in LKB1-depleted cells HUVEC were transfected with control-siRNA (siControl) or siRNA against LKB1 (siLKB1, 0.5 μg/ml 72 h). Thereafter, cells were treated with MK-8722 at indicated concentrations for 24 h. Cells were lysed and subjected to western blot analyses of LKB1 (**A**), AMPK phosphorylated at T172 (**B**), ACC phosphorylated at S79 (**C**), Raptor phosphorylated at S792 (**D**), and p70S6K phosphorylated at T389 (**E**), and the corresponding non-phosphorylated proteins or β-actin. Representative immunoblots and quantification by densitometry with normalisation of phosphorylated proteins to total proteins or to β-actin (LKB1) are shown. Mean + SD, *n* = 5. *P<0.05 versus untreated control, #*P<*0.05 versus control siRNA-treated samples stimulated with an equimolar dose of MK-8722. Two-way repeated measurement ANOVA corrected by the Holm–Šidák method was applied.

Since MK-8722 led to ATP depletion in endothelial cells (Figure 3D), we suggested that it may trigger indirect activation of AMPK in addition to the ADaM site-mediated allosteric activation. In case of energy depletion, AMP binds to the γ-subunit of AMPK, thereby promoting LKB1-mediated phosphorylation of T172. We found that down-regulating LKB1 with siRNA prevented AMPK phosphorylation induced by 10 μM MK-8722 (Figure 6A,B), indicating that this activation was triggered by ATP depletion. However, inhibiting AMPK phosphorylation in LKB1-depleted cells did not result in reduced phosphorylation of ACC or Raptor, and a decrease in p70S6K phosphorylation was still observed when the cells were treated with 10 μM MK-8722 (Figure 6C–E). This indicates that the LKB1-mediated activation was not functionally relevant. When cells were stimulated with lower concentrations of MK-8722 (0.1 and 1 μM), slightly reduced ACC and Raptor phosphorylation were observed (Figure 6C,D). Thus, the LKB1 pathway may become relevant at lower concentrations of MK-8722. The possibility that CaMKK2-mediated AMPK activation plays a role in this context was ruled out, as we did not detect any MK-induced increase in mitochondrial or cytosolic Ca^2+^ levels (see Supplementary Figure S5).

### Inhibition of ACC1 and mTORC1 by MK-8722 correlates with its antiviral effect against herpes simplex virus-1

**Figure 7:**
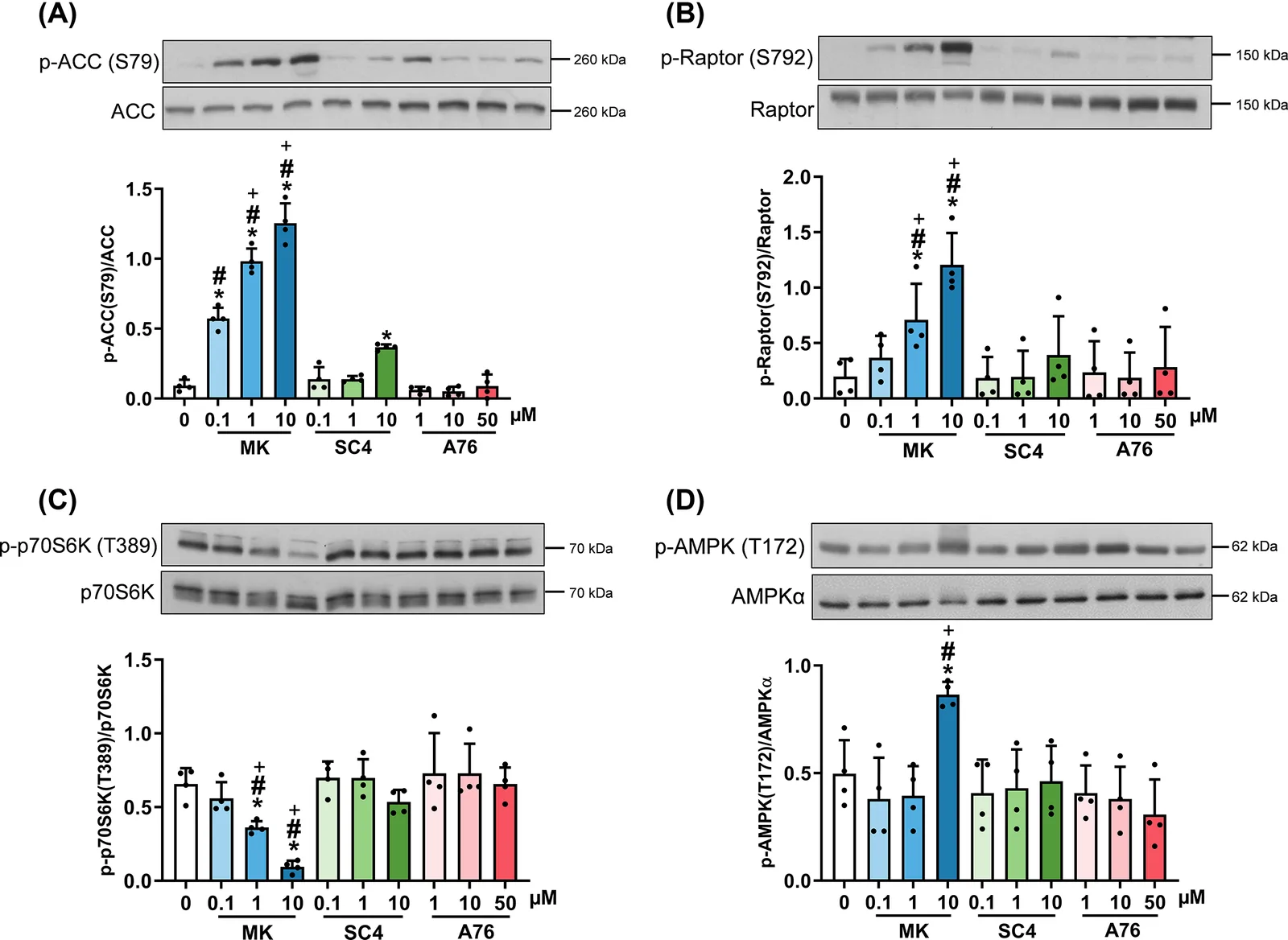
Activation of the AMPK pathway by MK-8722, SC4, and A-769662—long-term treatments in full growth medium HUVEC were treated with the MK-8722 (MK), SC4, and A-769662 (A76) at the indicated concentrations for 24 h in full growth medium. Cells were lysed and subjected to western blot analyses of ACC phosphorylated at S79 (**A**), Raptor phosphorylated at S792 (**B**), p70S6K phosphorylated at T389 (**C**), AMPK phosphorylated at T172 (**D**), and the corresponding non-phosphorylated proteins. Representative immunoblots and quantification by densitometry with normalisation of phosphorylated proteins to total proteins are shown. Mean + SD, *n* = 4. *P<0.05 versus untreated control, ^#^*P<*0.05 versus equimolar doses of SC4, ^+^*P<*0.05 versus equimolar doses of A-769662 by one-way repeated measurement ANOVA corrected by the Holm–Šidák method.

We have recently demonstrated the potent inhibitory effect of MK-8722 on HSV-1 replication in endothelial cells and identified AMPK as a potential target for antiviral strategies against HSV-1 infection [16]. To ascertain the functional relevance of the different AMPK activator profiles outlined above, we compared the effects of MK-8722, SC4, and A-769662 in our model of HSV-1 replication in endothelial cells. As viral infection studies are performed in full growth medium containing 20% serum, we first tested the effects of the compounds (concentrations as indicated, 24 h) on the AMPK pathway in such a medium (Figure 7). The activation patterns observed were similar to those described in a medium containing 2% fetal calf serum (FCS) (see Figure 2). MK-8722 induced increases of up to 13- and 5.5-fold, respectively, in the phosphorylation of ACC and Raptor compared with the untreated control, and decreased p70S6K phosphorylation by 90%. SC4 and A-769662 induced ACC phosphorylation to a much lesser extent, with almost no effect on Raptor phosphorylation observed. Accordingly, they did not inhibit p70S6K phosphorylation.

**Figure 8:**
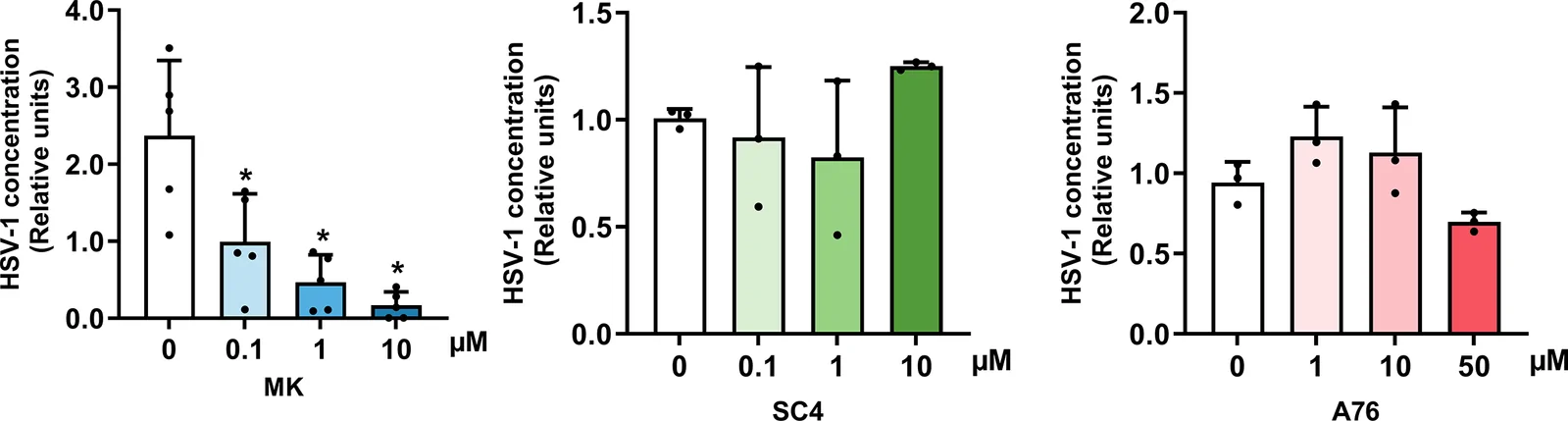
MK-8722 restricts HSV-1 replication in endothelial cells HUVEC were pretreated with MK-8722 (MK), SC4, and A-769662 (A76) at the indicated concentrations for 1 h and infected with HSV-1 for 24 h. HSV-1 concentration was determined by the TCID_50_ assay. For every independent experiment, the batch mean of virus titer was calculated from all the conditions, including controls and MK/SC4/A76 treatments, and each value was expressed relative to the batch mean. Mean + SD are demonstrated, *n* = 3–5. *P<0.05 versus untreated control by one-way repeated measurement ANOVA.

We then pre-treated endothelial cells with MK-8722, SC4, or A-769662 for 1 h before infecting them with HSV-1 for 24 h, after which we analysed virus levels in the cells and the cell culture medium. As shown in Figure 8, MK-8722 resulted in a strong, dose-dependent reduction in the number of virus particles, whereas SC4 and A-769662 had no effect. These data confirm that inhibiting HSV-1 replication via the AMPK pathway requires strong, long-lasting activation of the pathway, which was only achieved with MK-8722.

## Discussion

AMPK plays a crucial role in maintaining endothelial homeostasis. This encompasses energy metabolism, barrier function, vascular tone, angiogenesis, and protection against inflammatory and oxidative stress, as well as providing antiviral defence [14–16,34–39]. Therefore, targeting AMPK in endothelial cells could be an effective therapeutic approach for treating endothelial dysfunction, which often marks the beginning in the development of cardiovascular diseases [1–3]. It is therefore important to identify compounds that can efficiently activate AMPK in these cells. In the present study, we characterise three pharmacological AMPK activators, MK-8722, SC4, and A-769662, in primary human vein endothelial cells. We show that the pan-AMPK activator MK-8722, compared with SC4 and A-769662, is a highly potent AMPK activator in these cells. This was demonstrated through the phosphorylation kinetics of AMPK substrates and the antiviral activity of MK-8722, which highlights the functional relevance of our observations.

The three activators were tested at different concentrations and in various settings, including the acute addition of the activators to cells and chronic treatment in both serum-poor and serum-rich media. We used AMPK-specific phosphorylation of ACC and Raptor as well as the phosphorylation state of p70S6K, a downstream target of mTORC1, as read-outs for AMPK activation, since it is known that activation of AMPK by MK-8722, SC4, or A-769662 occurs independently from T172 phosphorylation [30,32,33]. The applied concentrations (0.1–10 μM for MK-8722 and SC4, and 1–50 μM for A-769662) were similar to those previously used *in vitro* as well as to the plasma levels observed in animal studies [28,29,31–33] and did not affect cell viability. MK-8722 was found to be the most potent AMPK activator of the tested compounds in all settings and on an equimolar basis.

The high AMPK-activating potency of MK-8722 may be related to its EC_50_ value. When tested with purified AMPK complexes containing the β1 isoform, MK-8722 exhibited the lowest EC_50_ value of the three compounds [28,32,33]. In addition, MK-8722 is known to permeate cells efficiently [32], which may contribute to its effectiveness. However, differences between the three agonists were also observed following long-term treatment, when differences in cell permeability may play a lesser role. Interestingly, we found that, at a concentration of 10 μM, MK-8722 decreased mitochondrial ATP production, maximal respiration, and spare capacity, whereas SC4 and A-769662 had no effect. This finding is consistent with a previous study demonstrating a dose-dependent suppression of the mitochondrial function in HEK293T/17 cells by MK-8722 [40]. Therefore, MK-8722 may activate AMPK via an energy depletion-dependent mechanism in addition to its allosteric effect, which could increase its AMPK-activating potency. Consistent with this, a 10 μM concentration of MK-8722 induced an LKB1-mediated AMPK activation, which is known to be triggered by energy depletion. However, LKB1 down-regulation had little impact on AMPK target phosphorylation under these conditions. This indicates that the direct allosteric activation of AMPK by MK-8722 via the ADaM site is already sufficient to fully stimulate AMPK-dependent pathways at a concentration of 10 μM. When lower concentrations of MK-8722 (0.1 or 1 μM) were applied, moderate effects of LKB1 down-regulation on AMPK target phosphorylation were observed. Therefore, at lower concentrations of MK-8722, the combined activation of AMPK via both direct and indirect mechanisms may play a role, even though energy depletion was not detectable under these conditions. Together, these data suggest that an additional activation mechanism of MK-8722, involving energy depletion and subsequent AMPK phosphorylation, may contribute to AMPK activation under certain conditions, but it does not fully explain its higher potency in activating AMPK.

The main reason for the strong activation of AMPK by MK-8722 in endothelial cells is likely to be related to the composition and functional significance of different AMPK heterotrimers in these cells. The predominant catalytic subunit in endothelial cells is known to be AMPKα1, while AMPKα2 is expressed to a lesser extent, yet still exerts significant effects on endothelial homeostasis [5]. The two subunits seem to be located in different parts of the cells since the AMPKα2 but not AMPKα1 contains a nuclear localisation sequence [41,42]. Our own data confirm the dominance of the AMPKa1 isoform at the mRNA level (Supplementary Figure S6). Additionally, we have demonstrated that the β1 and β2 and the γ1 and γ2 isoforms are all expressed at reasonable levels in endothelial cells, while the γ3 isoform was not detectable (Supplementary Figure S6). Thus, although the α1,β1,γ1 complex was previously assumed to be the major AMPK complex in endothelial cells [43], other heterotrimers may be expressed. In line with this, an α2,β2,γ2 AMPK complex functionally linked to mitosis was found in nuclear fractions of HUVEC [44], indicating that specific AMPK subunit activation may be linked to distinct cellular processes. Therefore, full AMPK activation in endothelial cells may require agonists that act as pan-AMPK activators, such as MK-8722, and the high potency of MK-8722 observed in our study may be, at least in part, explained by its broad specificity for several AMPK heterotrimers. Consistent with this, we have demonstrated that activation of AMPK by a physiological agonist, vascular endothelial growth factor, requires both AMPKα1 and AMPKα2 complexes [13].

To investigate whether the AMPK activation profiles of the tested compounds are associated with a protective function of AMPK, we compared the antiviral capacities of MK-8722, SC4, and A-769662. Previously, we demonstrated that AMPK controls HSV-1 replication in endothelial cells [16], whereby down-regulating the AMPKα1 and/or AMPKα2 catalytic subunits increased HSV-1 replication, and AMPK activation by MK-8722 strongly inhibited it. MK-8722’s antiviral effect was related to its strong and persistent phosphorylation of AMPK’s downstream targets, ACC and Raptor, which led to impairments in ACC1-mediated lipid synthesis and the mTORC1 pathway, respectively. Both of these are required for efficient HSV-1 replication [16]. Here, we confirmed strong AMPK activation in response to a 24 h incubation with MK-8722 in a serum-rich medium, which was necessary for studying virus replication. By contrast, SC4 and A-769662 induced only minor ACC and Raptor phosphorylation, respectively, under these conditions. Their effects in serum-containing medium were even lower compared with serum-poor medium, suggesting that scavenging effects of serum proteins may play a role. The impact of the three compounds on HSV-1 replication in endothelial cells was consistent with their respective AMPK activation profiles. Our data confirm the strong antiviral capacity of MK-8722, whereas SC4 and A-769662 had no impact on HSV-1 replication. As discussed above, different AMPK heterotrimers may be involved in the antiviral function of AMPK, which may only be activated by pan-AMPK activators, such as MK-8722. In line with this, we have recently shown that the antiviral activity of AMPK depends on both AMPKα1 and α2, indicating that distinct AMPK complexes are involved [16].

Although being a strong AMPK activator, the metabolic side effects of MK-8722 need to be considered when MK-8722 is experimentally employed. Our data show that MK-8722 at a concentration of 10 μM induces a mild mitochondrial dysfunction characterised by increased levels of mitochondrial and cytosolic ROS while mitochondrial permeability or morphology was not altered. Using isolated rat mitochondria, we found that MK-8722 inhibits the activity of respiratory chain complex I. This may explain its inhibitory effect on mitochondrial respiration and the increased ROS formation independently of AMPK activation. We noted the structure of MK-8722 shares striking similarities with potent complex I inhibitors IACS-010759 and BAY87-2234, which are proposed to occupy the hydrophobic ubiquinone-binding channel in the complex I subunit ND1 to block electron transfer from Fe-S clusters [45,46]. Therefore, at high concentrations, MK-8722 may also be able to access this channel to inhibit complex I function in a similar manner. Interference with mitochondrial energy metabolism adds to the known side effects of MK-8722 [32] and suggests that its use in cultured endothelial cells and possibly other models should be restricted to concentrations of ≤1 μM. SC4 did not affect mitochondrial function, whereas A-769662 moderately inhibited complex I activity and increased cytosolic ROS at high concentrations. As these concentrations were also necessary for significant AMPK activation, A-769662 appears to be an ineffective AMPK agonist in endothelial cells. However, since our data were obtained in HUVEC, i.e., in cells of venous origin derived from a foetal tissue, they need to be validated in other models that reflect the heterogeneity of endothelial cells across the vasculature.

In summary, we investigated the effects of the AMPK activators MK-8722, SC4, and A-769662 on endothelial cells by examining the phosphorylation of their downstream targets ACC and Raptor and analysing HSV-1 replication. Of the tested compounds, MK-8722 was found to be the most potent AMPK agonist, most likely due to its allosteric, pan-AMPK-activating properties. We show that MK-8722 at a concentration of 10 μM interferes with mitochondrial respiration by inhibiting complex I, leading to reduced mitochondrial ATP formation and increased ROS production. This may cause side effects but appears to play only a minor role as an additional mechanism for AMPK activation.

Our data suggest that pan-AMPK activators may be required to target endothelial dysfunction, whereas β1-selective activators or intermediate activators with a certain isoform preference may be less efficient. Nevertheless, further research into the distribution of heterodimers in endothelial cells and their specific functions is necessary to develop strategies for endothelial-targeted AMPK activation therapy.

## Materials and methods

### Chemicals

M199 medium was purchased from Capricorn (Ebsdorfergrund, Germany), Dulbecco’s Modified Eagle Medium, Nutrient mix F-12 (DMEM/F-12) from Life Technologies (Carlsbad, CA, U.S.A), and Eagle’s Minimum Essential Medium (EMEM) from Lonza (Basel, Switzerland). 4-(2-hydroxyethyl)-1-piperazineethanesulfonic acid solution (HEPES), FCS, human serum, endothelial cell growth supplement (ECGS), trypsin–EDTA, non-essential amino acids, L-glutamine, penicillin/streptomycin, human serum albumin (HSA), anhydrous dimethyl sulfoxide (DMSO), carbonyl cyanide 3-chlorophenylhydrazone (CCCP), and ionomycin. Hoechst 33342 and TBHP were from Sigma (Taufkirchen, Germany). Bovine serum albumin-C (BSA-C) was obtained from Aurion (Wageningen, The Netherlands), and goat serum from Cell Signaling Technology (Frankfurt, Germany). EDTA-free protease inhibitor cocktail was purchased from Roche Diagnostics (Mannheim, Germany). β-Mercaptoethanol, dithiothreitol (DTT), EDTA, and Tween-20 from Carl Roth GmbH (Karlsruhe, Germany). Triton X-100 was obtained from Farek Berlin (Berlin, Germany). Fluoromount-G® was from Southern Biotech (Birmingham, AL, U.S.A.). MK-8722 was purchased from Aobious (Gloucester, MA, U.S.A.) and A-769662 from Abcam (Cambridge, U.K.). SC4 was synthesised as previously described [33]. The siRNAs against LKB1 and non-targeting control siRNA were SMARTpool-siRNAs obtained from Horizon, Dharmacon RNAi, and Gene Expression (Lafayette, CO, U.S.A.), respectively.

### Antibodies

Rabbit monoclonal antibodies for western blotting against β-actin (#4970), AMPKα (#2532), ACC1 (#3676), p70S6K (#9202), Raptor (#2280), LKB1 (#3079), and against phospho-AMPK (T172) (#2535), phospho-ACC (S79) (#3661), phospho-p70S6K (T389) (#9205), and phospho-Raptor (S792) (#2083) were obtained from Cell Signaling Technology (Frankfurt, Germany) and were used at 1:1000 dilutions. Rabbit recombinant Anti-TOMM20 antibody [EPR15581-54] (#ab186735) and mouse monoclonal Anti-ATPB antibody [3D5] (#ab14730) were obtained from Abcam (Cambridge, U.K.). Peroxidase-labeled goat anti-rabbit IgG was from Kirkegaard and Perry Laboratories, Inc. (Gaithersburg, MD, U.S.A.). Secondary AlexaFluor®488-conjugated goat anti-rabbit IgG and goat anti-mouse IgG were from Thermo Fisher Scientific (Waltham, MA, U.S.A.).

### Cells

To prepare HUVEC, umbilical cord veins were washed with 0.9% NaCl solution and incubated at 37°C for 3 min with 0.01% collagenase dissolved in M199. After rinsing the veins with M199/10% FCS, the detached cells were centrifuged at 500×***g*** for 6 min. The resulting pellet was resuspended in M199/10% FCS, after which the cells were seeded onto a cell culture flask that had been coated with 0.2% gelatin. After 24 h, cells were washed and cultured further in full growth medium (M199, 17.5% FCS, 2.5% human serum, 7.5 μg/ml ECGS, 7.5 U/ml heparin, 680 μM glutamine, 100 μM vitamin C, 100 U/ml penicillin, 100 g/ml streptomycin) at 37°C and 5% CO_2_. For most experiments, cells from the second passage were seeded at a density of 27,500/cm^2^ and used for experiments three days after seeding. For siRNA transfection, the seeding density was 23,000/cm^2^. Immunofluorescence studies were performed with primary endothelial cells seeded on coverslips (70,000–100,000 cells/cm^2^). Most experiments were performed with cells on 30-mm culture dishes. For experiments involving lentiviral transduction, HUVEC from the first passage were used and seeded onto 12-well plates at a density of 10,000 cells/cm^2^ 24 h prior to infection. For calcium measurements, HUVEC transduced with lentivirus were seeded onto coverslips at a density of 140,000–200,000 cells/cm^2^.

### Cell stimulation

The activation profiles of the AMPK activators MK-8722, A-769662, and SC4 were compared. The respective compounds were dissolved and diluted in DMSO, and an equal amount of solvent was added to untreated cells for each condition. To study the AMPK-activating properties, cells were incubated with different concentrations of the respective AMPK agonist in either medium containing 2% FCS (M199, 2% FCS, 680 μM glutamine, 100 U/ml penicillin, 100 g/ml streptomycin) for 15 min or 24 h or in full growth medium for 24 h. In the first case, the 2% FCS medium was added to the cells 2 h prior to stimulation with the AMPK activators.

### Western blot analysis

The endothelial cells were lysed on ice by a 15 min incubation in 50 mM Tris buffer (pH 7.4) containing 2 mM EDTA, 1 mM EGTA, 50 mM NaF, 10 mM Na_4_P_2_O_7_, 1 mM, Na_3_VO_4_, 1 mM DTT, 1% Triton X-100, 0.1% SDS, 1 mM PMSF, and 10 μl/ml protease inhibitor cocktail. The lysates were then centrifuged at 700×***g*** for 6 min, after which aliquots were used to determine the protein concentration using the DC^™^ Protein Assay kit (Bio-Rad, Feldkirchen, Germany). The remaining supernatant was supplemented with Laemmli buffer, subjected to SDS–PAGE (30–50 μg of lysate protein/lane), and transferred onto polyvinylidene fluoride membranes. The membranes were blocked for 1.5 h in Tris-buffered saline/Tween (TBST) (20 mM Tris (pH 7.6), 137 mM NaCl, 0.1% (v/v) Tween® 20) containing 5% non-fat dried skimmed milk. Incubations with primary antibodies diluted in TBST containing 5% BSA were then performed overnight at 4°C. The following day, the membranes were incubated with the respective horseradish peroxidase-conjugated secondary antibodies for 1 h. Signal detection was performed using either the enhanced chemiluminescence (ECL) reagent (GE Healthcare, Chicago, IL, U.S.A.) or the Western Lightning Plus-ECL reagent (Perkin Elmer, Waltham, MA, U.S.A.). Quantification of protein bands was carried out by densitometry using ImageJ software (1.52P, National Institutes of Health, U.S.A.). For expression studies, the ratios of the protein of interest to β-actin were calculated. Signals of phosphorylated proteins were normalised to the respective total protein signals.

### Seahorse analyses of cells

HUVEC were seeded onto Seahorse XF96 Cell Culture Microplates (5000 cells/well; Agilent Technologies, Santa Clara, CA, U.S.A.) and incubated with AMPK activators for 24 h in 2% FCS medium. For the Mito stress test, the incubation or culture medium was replaced with Seahorse XF Base Medium (103334-100, Agilent Technologies, pH adjusted to 7.4), supplemented with 10 mM D-glucose, 2 mM L-glutamine, and 1 mM sodium pyruvate. The cells were then cultured for another hour in a CO_2_-free incubator at 37°C. In experiments without pretreatment, MK-8722, SC4, or A-769662 were added directly to the Seahorse medium.

OCR was monitored at basal conditions as well as after sequential injection of 2 μM oligomycin to block the mitochondrial ATP synthase, 2 μM FCCP to uncouple oxidative phosphorylation, and 2 μM antimycin A to fully inhibit mitochondrial respiration. Measurements were performed in 3 min mix and 3 min measure cycles at 37°C in six replicates per condition using a Seahorse XFe96 Analyzer (Agilent Technologies). OCR was depicted as pmol/min and normalised to the exact cell number of each well, which was determined by high-content microscopy. Wave software (Agilent Technologies) was used to analyse the datasets.

### siRNA treatment

Endothelial cells were seeded in 30-mm dishes at 50–70% confluency one day before transfection. Transfection was performed using the SAINT-sRNA transfection kit (Synvolux Therapeutics B.V., Leiden, The Netherlands) with 0.5 μg/ml non-targeting or specific siRNA. For each 30-mm dish, the siRNA was diluted in 100 μl of phosphate-buffered saline (PBS), combined with 20 μl of Saint-sRNA diluted in 80 μl of PBS, and then supplemented with 800 μl of M199 containing 0.25% HSA. The cells were washed twice with pre-warmed Hanks’ balanced salt solution before the transfection solution was added. After 4 h, 2 ml of growth medium was added, and the cells were cultured for 72 h before experiments were performed. The down-regulation efficiency for the targeted proteins was verified by western blotting.

### Isolation of heart mitochondria

Cardiac mitochondria from adult rats (23–37 weeks old) were isolated by differential centrifugation, as previously described [47]. Animal procedures were approved by the responsible local authorities (animal welfare office of the UKJ (University Hospital Jena), Thüringer Landesamt für Verbraucherschutz) and registered as TWZ-19-2020. Animals were killed with cervical dislocation under deep anesthesia (sodium thiopental 300 mg/kg) at University Hospital Jena. Interfibrillar (IFM) and subsarcolemmal (SSM) mitochondria were prepared from freshly explanted heart muscle (excluding both atria) using a modified Chappell–Perry buffer (containing 100 mM KCl, 50 mM MOPS, 1 mM EGTA, 5 mM MgSO_4_·7H_2_O, and 1 mM ATP, pH 7.4, 4°C). To release IFM from the myofibrillar network, homogenates were treated with trypsin (5 mg of trypsin per g wet weight of cardiac tissue) for 10 min at 4°C. After separation, IFM and SSM were pooled (*n* = 4) and resuspended in KME buffer (100 mM KCl, 50 mM MOPS, 0.5 mM EGTA, at pH 7.4). Mitochondrial protein concentration was determined by the Bradford method using bovine serum albumin as a standard (Protein Assay Dye Reagent Concentrate, Bio-Rad; protein standard, Sigma).

### Determination of isolated complex activities

The freshly isolated cardiac mitochondria (IFM/SSM pool) were treated with 1 mg cholate/mg mitochondrial protein and then further prepared according to [48]. After one cycle of freeze/thaw using temperatures of −80°C or 25°C, respectively, the activities of the electron transport chain complexes were measured as specific donor-acceptor oxidoreductase activities, as previously described [49]. Each mitochondrial preparation (*n* = 4) was incubated immediately prior to measurement with activators. Complex I was measured as rotenone-sensitive reduction of 2,6-dichloroindophenol using NADH as the substrate [50]. Reduction of 2, 6-dichloroindophenol using succinate as the substrate was used to assess complex II [51]. Complex III activity was determined by measuring the antimycin-A-sensitive reduction of cytochrome c [51] using decylubiquinol as the substrate [52]. Complex IV activity was determined by the oxidation of reduced cytochrome c [53]. Complex V (F_0_F_1_-ATPase) activity was assessed in the reverse direction by measuring ATP hydrolysis coupled to the oxidation rate of NADH [54].

### Mitochondrial and cellular ROS Measurement

Mitochondrial ROS levels were detected using the MitoSOX Red mitochondrial superoxide indicator, and cellular ROS levels were measured using CM-H_2_DCFDA, both from Thermo Fisher Scientific (Waltham, MA, U.S.A.). After treatment for 24 h in 2% FCS medium, the cells were washed once with PBS and then incubated with the staining solution (3 μM MitoSOX™ or 5 μM CM-H2DCFDA, respectively, in HEPES buffer (10 mM HEPES (pH 7.4), 145 mM NaCl, 5 mM KCl, 1 mM MgSO_4_, 1.5 mM CaCl_2_, 10 mM glucose, 0.25% HSA) for 30 min. CCCP (100 μM, 30 min), a mitochondrial uncoupler, was added as a positive control for measuring mitochondrial ROS, and TBHP (150 μM, 30 min) was used as a positive control for measuring cellular ROS. Both reagents were added during the incubation of the cells with the respective staining solutions. Following incubation with MitoSOX™ or CM-H2DCFDA, respectively, the cells were detached using Trypsin/EDTA and centrifuged at 500×***g*** for 1 min. The cell pellets obtained were resuspended in PBS and subjected to flow cytometry analysis using the LSR Fortessa (BD Biosciences, Heidelberg, Germany) with the Diva software. The 488 nm laser with the 695/40 bandpass filter, PerCP-Cy5-5-A, was used for MitoSOX, and the 488 nm laser with the 530/30 bandpass filter, Alexa Fluor® 488, was used for CM-H2DCFDA. Median values were obtained using the FlowJo™ v10.7.2 software (BD Biosciences, Franklin Lakes, NJ, U.S.A.). The unstained values were subtracted from each individual value for each batch before analysis.

### Mitochondrial membrane potential using JC-10

Mitochondrial membrane potential was measured using the JC-10 Mitochondria Labeling Reagent from Thermo Fisher Scientific (Waltham, MA, U.S.A.). This reagent allows healthy and damaged mitochondria-containing cells to be differentiated according to the fluorescent intensities in the PE and FITC channels, respectively. Following treatment for 24 h in 2% FCS medium, the cells were washed once with PBS and incubated with the staining solution (10 μM JC-10 in HEPES buffer) for 30 min. CCCP (100 μM, 30 mins), a mitochondrial uncoupler, was added as a positive control during the incubation of cells with JC-10. Cells were then detached using Trypsin/EDTA and centrifuged (500×***g***, 1 min). The obtained cell pellets were resuspended in PBS and analysed using flow cytometry performed on a FACS Celesta (BD Biosciences, Heidelberg, Germany) using Diva software. The 488 nm laser with the 505 and 550 long pass filter was used with the 530/30 (FITC channel—damaged mitochondria) and 610/20 (PE channel—healthy mitochondria) bandpass filters, respectively. Median values were obtained using the FlowJo™ v10.7.2 software (BD Biosciences, Franklin Lakes, NJ, U.S.A.). The unstained values were subtracted from each individual value for each batch before analysis.

### Immunofluorescence

After treatment of endothelial cells for 24 h in 2% FCS medium, the cells were washed with warm (37°C) PBS, fixed with 4% paraformaldehyde for 15 min, washed twice with PBS, and permeabilised in PBS containing 0.1% Triton X-100 for 5 min. After two further washes with PBS, the cells were incubated with a blocking solution containing 5% goat serum and 1% BSA-C in PBS for 30 mins. After washing the cells twice with PBS, they were incubated with the primary antibody for 1 h (1:500 for TOMM20 and 1:1000 for ATPB), followed by the conjugated secondary antibody (1:1000) for 1 h, with two PBS washing steps in between. Following two PBS washes, the cells were incubated with Hoechst 33342 (1 μg/ml in PBS) for 10 min. After three PBS washes and one H_2_O wash, the coverslips were mounted on microscopic slides using Fluoromount-G. Immunofluorescence images were acquired using a LEICA DMi8 TCS SP8 inverted laser-scanning microscope operated with the Leica Application Suite X (Leica Biosystems, Wetzlar, Germany). Images in a 1024 × 1024 pixel format were acquired using an HC PL APO CS2 63x/1.40 oil objective with laser excitation at 488 nm and a scan speed of 400 Hz. Image acquisition was single-blinded and randomised, with areas of cells scanned primarily in brightfield illumination to avoid bias and unintentional photobleaching. Images were analysed using ImageJ software (1.52P, National Institutes of Health, U.S.A.).

### Virus

The HSV-1 strain KOS [55] (kindly provided by the Section for Experimental Virology, Institute of Medical Microbiology, Jena University Hospital) was used. Virus propagation and titration experiments were carried out with African Green Monkey Kidney cells (GMK, Vero, ATCC number: CCL-81), cultured at 37°C and 5% CO_2_ in EMEM containing 10% FCS, 1% non-essential amino acids, 100 U/ml penicillin, 100 mg/ml streptomycin, and 2 mM L-glutamine. 100,000 cells were seeded in each well of a 96-well plate and allowed to adhere for 1 h, after which the TCID_50_ assay was performed to determine viral replication.

### Virus infection and titration

Confluent endothelial cells in 24-well plates were pretreated with AMPK activators for 1 h in complete growth medium. The cells were then infected with the HSV-1 KOS strain for 1 h under serum-free conditions, at a multiplicity of infection of 5 alongside untreated controls. One hour post-infection, the virus-containing medium was replaced with fresh full growth medium containing the activators, after which the cells were incubated for a further 24 h. After this period, the plates were frozen at −20°C for one day and then thawed at room temperature to release the intracellular viruses into the medium. The samples containing both intracellular and extracellular viruses were briefly centrifuged at 20,000×***g***. The obtained supernatants were serially diluted and transferred onto GMK reporter cells to determine the respective amounts of virus present using the TCID_50_ assay. Positive cytopathic effects correlating with the amount of virus present were documented and used to calculate the virus titer according to the Reed and Muench method [56]. Each experimental condition was tested in triplicate. To correct for the variability between biological replicates, the virus titers were normalised against the mean value of each cell batch calculated from untreated and treated conditions of the respective batch. The relative values from each biological replicate were then used to calculate the mean of control and treated samples of all replicates as shown in Figure 8.

### Cell viability using cell counting Kit-8

Cell Counting Kit-8 (CCK8; Sigma-Aldrich, #96992) assay was used to analyse the cell viability. Endothelial cells were seeded at a density of 5000 cells per well in a 96-well plate and cultured overnight. After 24 h of treatment of the activators in 2% FCS medium, 10 μl of CCK-8 solution was added to each well, and the plate was incubated at 37°C for 2 h. The absorbance was then measured at 450 nm using a spectrophotometer (Sunrise™) and the Magellan™ software. Cell viability was normalised to non-treated cells.

### Calcium measurements using cytosolic (GCaMP6f) and mitochondrial (Mitycam) sensors

#### Lentiviral transduction

The calcium sensors (pMOS008: GCaMP6F calcium sensor (cytosolic) and pMOS028: Mitycam calcium sensor (mitochondrial)) were ordered from Addgene (Watertown, U.S.A.), as detailed in [57]. The pMOS008: GCaMP6F calcium sensor was a gift from Adam Cohen (Addgene plasmid #163045; http://n2t.net/addgene:163045; RRID: Addgene_163045). The pMOS028: Mitycam calcium sensor was also a gift from Adam Cohen (Addgene plasmid #163046; http://n2t.net/addgene:163046; RRID: Addgene_163046).

For virus production, HEK293T cells were seeded in 90-mm dishes (26,000 cells/cm^2^) in 10 ml of DMEM/F-12 supplemented with 10% FCS, 2 mM glutamine, 100 U/ml penicillin, and 100 mg/ml streptomycin. The cells were then grown for 24 h. Co-transfection of HEK293T cells was performed with the lentiviral packaging plasmids pMDL (10 μg) and pRSV (5 μg), the envelope plasmid pVSVg (2 μg), and the plasmids of interest, GCaMP6F (10 μg) or MityCam (10 μg). The plasmids were mixed with 67.5 μl of polyethylenimine (1 μg/ml stock solution) in 1 ml of DMEM/F-12 without additives, incubated for 30 min at room temperature, and then added to the cells for 24 h. After this time, the virus-containing supernatants were harvested and sterile-filtered (0.2 μm) before being concentrated approximately 30-fold using Amicon centrifugal filter tubes. Supernatants from four dishes were pooled per condition. HUVEC were seeded in a 12-well plate 24 h prior to lentiviral infection. For transduction, 15 μl/1.5 ml of a 0.8 mg/ml stock solution of polybrene was added per well, followed by the equal distribution of the viral concentrate onto the cell layer. The plates were then centrifuged for 1 h at 500×***g***, and the infection was repeated after 24, 48, and 72 h. Fresh full growth medium was added to the cells after 4–5 h of the final infection. After 3–5 days, the confluent infected HUVEC were seeded onto 24-mm coverslips that had been double-coated with 1% gelatin for the microscopy experiment.

#### Live-cell imaging of cytosolic calcium using CGaMP6F calcium sensor

Infected HUVECs were incubated in 2% FCS medium for 2 h prior to live-cell imaging. For single-cell imaging, each coverslip was mounted in an Attofluor imaging chamber (Thermo Fisher Scientific, Germany) containing HEPES buffer as the imaging medium. Imaging was performed using an inverted laser scanning confocal microscope (DMi8 TCS SP8, Leica Microsystems) equipped with an HC APO CS2 63×/1.40 oil objective (Leica). Cells were excited with a 488 nm argon laser, and fluorescence emission was collected in the 494–560 nm range. Time-lapse images were acquired every 500 ms. The imaging protocol began with recording basal fluorescence in HEPES buffer, followed by addition of either 10 μM MK-8722 or DMSO in HEPES buffer (control), and concluded with 1 μM ionomycin as a positive control to elicit a maximal fluorescence response for normalisation purposes. For analysis, regions of interest (ROIs) were drawn around individual cells and a background area devoid of cells and fluorescence for each coverslip. Fluorescence intensities were extracted, and changes in fluorescence before and after reagent or ionomycin addition were calculated after subtracting background fluorescence. The average fluorescence of all cells within each coverslip was then normalised to the ionomycin response, which was set to 100%.

#### Live-cell imaging of mitochondrial calcium using Mitycam calcium sensor

Prior to live-cell imaging, infected HUVECs were incubated at room temperature in 2% FCS medium for 2 h prior to live-cell imaging. For single-cell imaging, each coverslip was mounted in an Attofluor imaging chamber (Thermo Fisher Scientific, Germany) containing HEPES buffer as the imaging medium. Imaging was performed using an inverted epifluorescence microscope (Axio Observer, Zeiss), which was equipped with an oil-immersion 63× objective lens (Plan-Apochromat 63×/1.4 Oil DIC III), an LED light source (CoolLED pE-4000), and an EMCCD camera (iXon Life 897, Andor). A superfast micro-manifold injection/perfusion system (Octaflow II V8, ALA Scientific Instruments) supplemented with a micro-manifold manipulator (Narishige, Japan) was used to deliver solutions during imaging. The cells were excited at 490 nm using a YFP filter cube (excitation: 500/20 nm, emission: 535/30 nm, long-pass: 515 nm). Imaging was controlled and monitored using VisiView software (version 4.5.01, Visitron Systems). Prior to image acquisition, ROIs were defined around individual cells and a background area (devoid of cells and fluorescence) for each coverslip. Time-lapse images were acquired every 100 ms under continuous perfusion flow. The imaging protocol consisted of sequential perfusion steps beginning with basal fluorescence recorded in HEPES buffer, followed by vehicle/mock stimulation with HEPES buffer containing DMSO. Next, cells were stimulated with 10 μM MK-8722 as the test reagent and finally with 100 μM CCCP to determine the maximal Ca^2+^ response and confirm the dynamic range of the sensor. For the analysis, the change in fluorescence intensity was calculated for each condition (vehicle/mock, MK-8722, and CCCP) by subtracting the baseline values from the endpoint values. Within each coverslip, the CCCP response from all cells was averaged. Responses to MK-8722 and mock stimulation were then normalised to this average CCCP response. Finally, the responses to MK-8722 and vehicle/mock were averaged across all cells within a coverslip and then averaged across all coverslips within a batch. The data are presented as a percentage of the maximal CCCP response (set to 100%).

### RNA sequencing

Confluent HUVECs in 90-mm culture dishes were lysed with 2 ml of TriReagent (Sigma-Aldrich, Taufkirchen, Germany). RNA extraction was performed by adding 200 μl of chloroform to 1 ml of sample. The sample was mixed, incubated for 7 min at room temperature, and centrifuged at 12,000×***g*** for 15 min at 4°C. The upper phase was then carefully transferred to a new tube, to which 500 μl of isopropanol was added to precipitate the RNA for 10 min at room temperature. After mixing and centrifugation at 12,000×***g*** for 10 min at 4°C, the RNA precipitate was washed once with 75% cold ethanol, followed by centrifugation at 7500×***g*** for 5 min at 4°C. The pellet was allowed to dry for 10 min, after which nuclease-free water was added and samples were incubated for 5 min at 57°C. The RNA content was measured using a Nanodrop spectrophotometer. RNA samples were sequenced using Illumina’s next-generation sequencing methodology [58]. In detail, total RNA was quantified and checked for quality using the Agilent 2100 Bioanalyzer Instrument (RNA 6000 Nano assay). Libraries were prepared from 1000 ng of total RNA using a TruSeq Stranded mRNA Library Preparation Kit (Illumina) according to the manufacturer’s instructions. The quantification and quality check of libraries was done using the Agilent 2100 Bioanalyzer Instrument (DNA 7500 assay). The libraries were then pooled and sequenced using a HiSeq 2500 System running in 51-cycle, single-end, rapid mode. The sequence information was then converted to FASTQ format using bcl2fastq v1.8.4. Read mapping to the reference was done using TopHat v2.1.0 [59] with the following parameters: -x 1, --no-coverage-search, --no-convert-bam, --no-novel-juncs, --no-novel-indels -T, --transcriptome-index=TRANSCRPTOME-INDEX. The human Ensembl genome version GRCh38 with annotation release 89 was used as the reference [60]. The number of mapped reads per gene was counted for each sample using featureCounts v2.0.3 [61] with the parameter -s 0. RPMs and RPKMs were then calculated based on gene counts using the R programming language (v4.1.3). Relative expression of the AMPK α1/α2, β1/β2, and γ1/γ2/γ3 subunit isoforms was calculated based on the RPKM values.

### RNA isolation, reverse transcription, and quantitative real-time PCR

Endothelial cells seeded on 30-mm dishes were lysed, and RNA was isolated using the column-based method by the NucleoSpin RNA Kit (Macherey-Nagel, Düren, Germany) as per the manufacturer’s protocol. Equal RNA concentrations from each sample were converted to cDNA by RevT-PCR using the First Strand cDNA Synthesis Kit by Thermo Fisher Scientific (Waltham, MA, U.S.A.) according to the manufacturer’s protocol. The cDNA samples were used for RT-qPCR, using the SYBR Green qPCR Master mix by Thermo Fisher Scientific (Waltham, MA, U.S.A.). The master mix consisted of buffer, thermostable DNA polymerase, deoxyribonucleotide triphosphates, and the SYBR Green dye. Each sample was measured in triplicates for each specific primer in a 96-well plate. The reaction mixture consisted of 10 μl of SYBR Green master mix, cDNA, and water and 2 μl of the specific primer pair in each well. PCR was performed for 30 cycles with 15 s at 95°C and 1 min at 60°C. The mean value of the triplicates per sample was normalised to the expression of the housekeeping gene (β-actin), and the percentage of each AMPK isoform was calculated from the total expression. All primer pairs used are enlisted in Table 1 and were purchased from Sigma (Taufkirchen, Germany).

**Table 1:** Primers for real-time PCR

Primer name	Sequence
β-actin	Forward: GGGACGACATGGAGAAAATCTG Reverse: GAAGGTCTCAAACATGATCTGGG
AMPKα1	Forward: TGTGATGGGATCTTCTATACC Reverse: CCCTGATATCTTTGATTGTGG
AMPKα2	Forward: ATGATGAAGTAGTGGAGCAG Reverse: CTTGATCTTGGTCTGTGTAAG
AMPKβ1	Forward: AAGGAGAGCATCAGTACAAG Reverse: ATGATGTTGTTAACTGTGCC
AMPKβ2	Forward: TTAAGGACAGTGTGATGGTC Reverse: CCTTCAAATGGGCTTGTATAG
AMPKγ1	Forward: GATGTTATCAATCTGGCAGC Reverse: CCACATCATTTTCATCCACC
AMPKγ2	Forward: TATGTCTGATATGCCAAAGC Reverse: CAGAGCTGATATTCGTCTTTC

### Statistical analysis

Information on the number of biological replicates used for each experiment is given in the figure legends. Significance was calculated using a one-way or two-way repeated measures ANOVA, after variance analysis, with Holm–Šidák correction for multiple comparisons using Sigmaplot 14.5. A *P*-value of less than 0.05 was considered significant. The data in the figures are presented as the mean + SD of independent experiments performed using cells from different donors.

## Supplementary Material

Supplementary Figures S1-S6

## Data Availability

The RNA sequencing data discussed in this publication will be deposited in NCBI’s Gene Expression Omnibus [62] and are accessible through GEO Series accession number GSE335379 (https://www.ncbi.nlm.nih.gov/geo/query/acc.cgi?acc=GSE335379).

## Abbreviations

ADaM: allosteric drug and metabolite
ANOVA: analysis of variance
ACC: acetyl-CoA carboxylase
AMPK: AMP-activated protein kinase
ATPB: ATP synthase beta subunit
BSA-C: bovine serum albumin-C
CaMKK2: calmodulin-dependent protein kinase kinase 2
CCCP: carbonyl cyanide m-chlorophenylhydrazone
DMEM/F-12: Dulbecco’s Modified Eagle Medium, Nutrient Mix F-12
DMSO: dimethyl sulfoxide
DTT: dithiothreitol
ECL: enhanced chemiluminescence
ECGS: endothelial cell growth supplement
EMEM: Eagle’s Minimum Essential Medium
FCCP: carbonyl cyanide-4-(trifluoromethoxy)phenylhydrazone
FCS: fetal calf serum
HEPES: 4-(2-hydroxyethyl)-1-piperazineethanesulfonic acid solution
HSA: human serum albumin
HSV-1: herpes simplex virus type 1
HUVEC: human umbilical vein endothelial cells
IFM: interfibrillar
LKB1: liver kinase B1
mTORC1: mechanistic target of rapamycin complex 1
OCR: oxygen consumption rate
PBS: phosphate-buffered saline
ROIs: regions of interest
ROS: reactive oxygen species
SSM: subsarcolemmal
T172: threonine 172
TBHP: tert-butyl hydroperoxide
TBST: Tris-buffered saline/Tween
TOMM20: translocase of outer mitochondrial membrane 20
