# Supplementary material for: The pan-AMPK activator MK-8722 outperforms the AMPK activators SC4 and A-769662 in human endothelial cells
Source: Biosci Rep. 2026 Sep 22;46(10):BSR20250408. doi: 10.1042/BSR20250408 (PMC13598317; doi:10.1042/BSR20250408)
Supplement: Supplementary file 1 [file BSR-2025-0408-T_supp.pdf]

The pan-AMPK activator MK-8722 outperforms the AMPK activators SC4 and A-769662 in human endothelial cells

Heena Doshi[1], Katrin Spengler[1], Kanstantsin Siniuk[2], Marco Groth[2], Estelle Heyne[3], Michael Schwarzer[3], Amod Godbole[1], Yi Sing Gee[4], Jonathan Baell[4,5], Jonathan S Oakhill[6,7], Andreas Henke[8], Regine Heller[1]

*Correspondence: regine.heller@med.uni-jena.de; Tel.: +49-3641-939 5633

Supplementary Figure 1

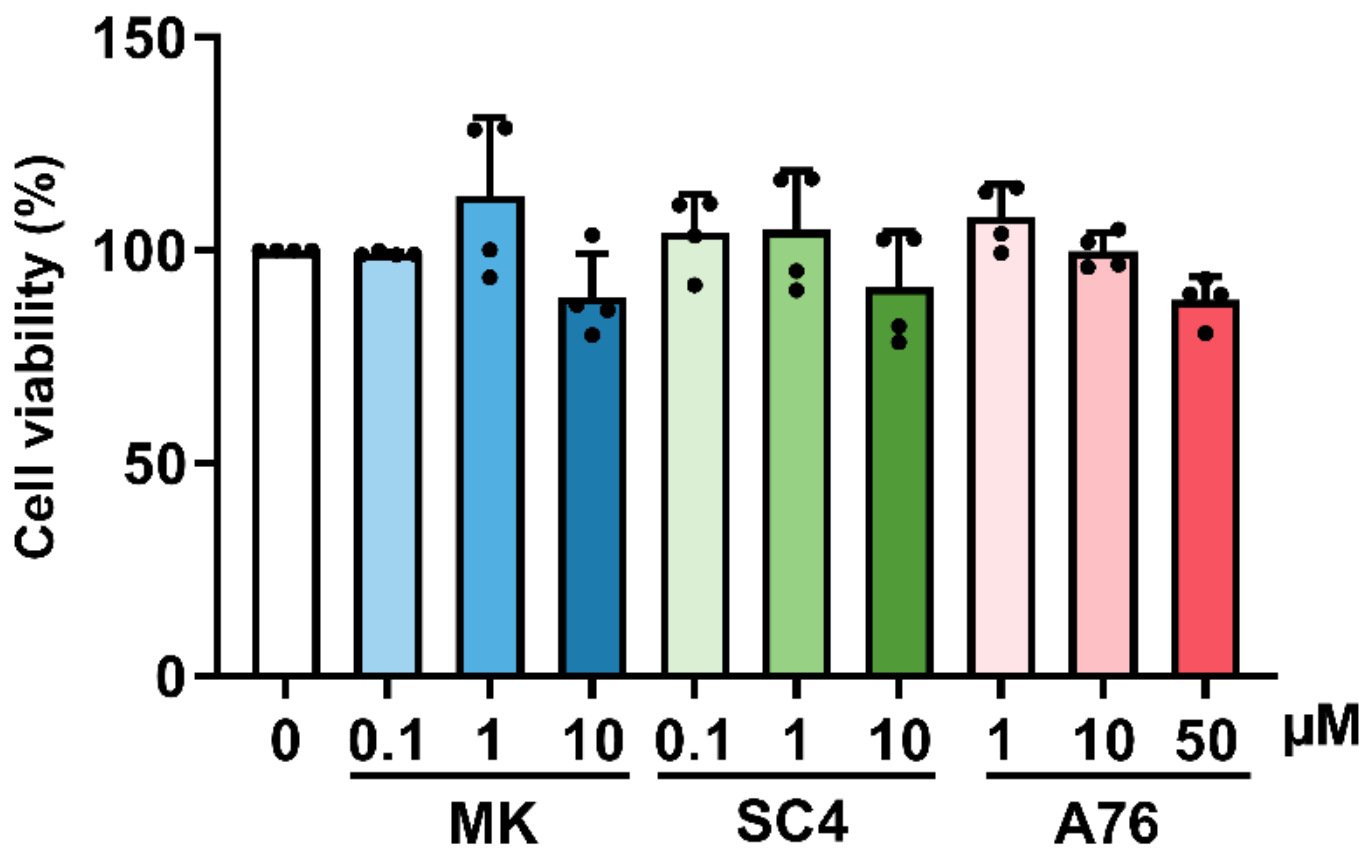

Supplementary Figure 1: Effect of AMPK activators on cell viability. HUVEC were treated with the MK-8722 (MK), SC4 and A-769662 (A76) at the indicated concentrations for 24 h. Cell viability was determined using the CCK-8 assay kit. Results were normalised to values obtained in non-treated cells. Mean + SD, n=3.

Supplementary Figure 2A

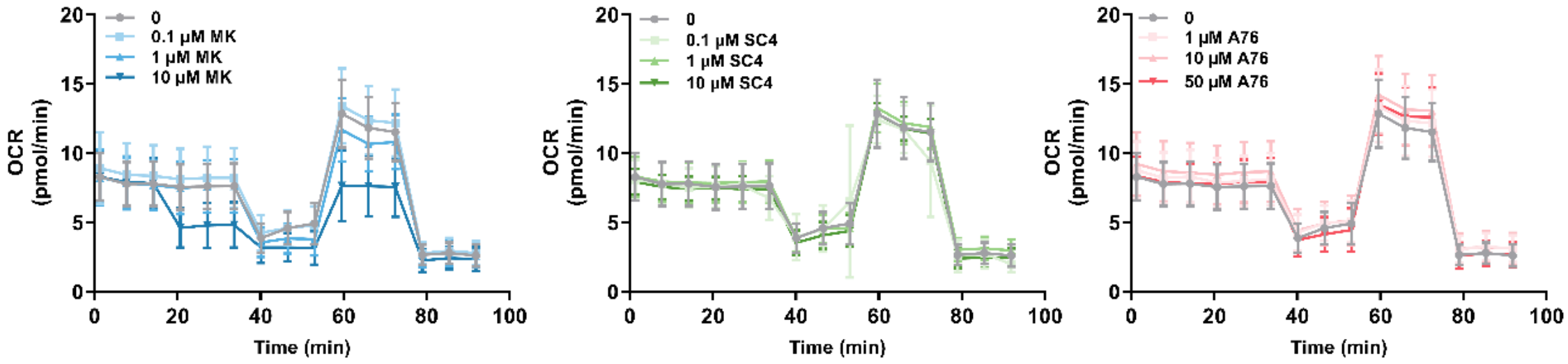

Supplementary Figure 2A: OCR trace with the acute additions of MK-8722, SC4 and A-769662. MK-8722 (MK), SC4 and A-769662 (A76) were added to the Seahorse assay medium at the indicated concentrations and oxygen consumption rate (OCR) was measured under basal conditions and following the sequential addition of compounds that modulate the respiratory chain: oligomycin to block the mitochondrial ATP synthase (2 µM), FCCP to uncouple oxidative phosphorylation (2 µM) and antimycin A (2 µM) to inhibit mitochondrial respiration. Mean ± SD, n=4.

Supplementary Figure 2B

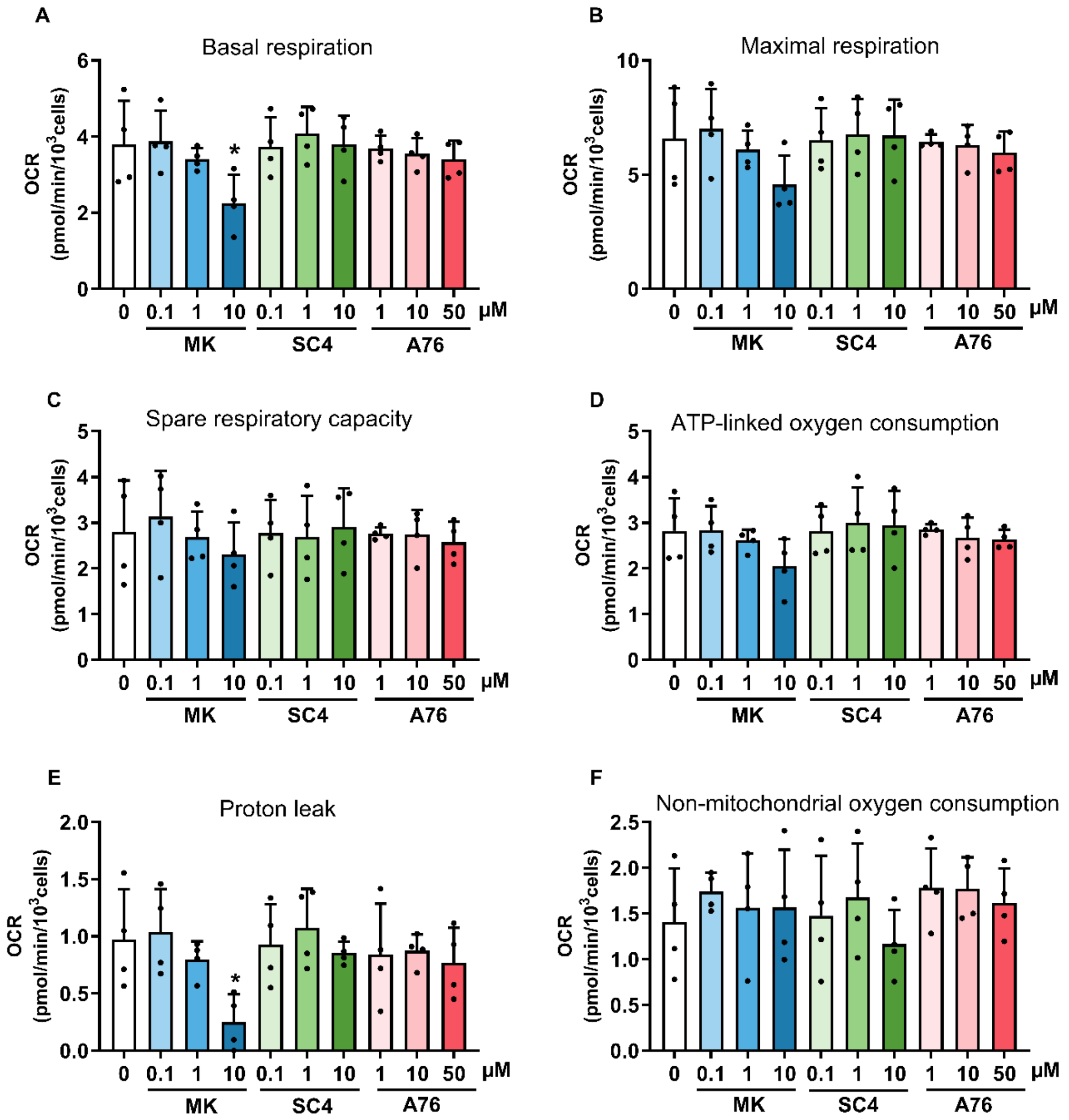

Supplementary Figure 2B: Effects of long-term treatment of endothelial cells with MK-8722 on mitochondrial energy metabolism. HUVEC were treated with the MK-8722 (MK), SC4 and A-769662 (A76) at the indicated concentrations for 24 h and then subjected to Seahorse analysis. Oxygen consumption rate (OCR) was measured under basal conditions and following the sequential addition of compounds that modulate the respiratory chain: oligomycin to block the mitochondrial ATP synthase, FCCP to uncouple oxidative phosphorylation and antimycin A to inhibit mitochondrial respiration. Parameters of mitochondrial metabolism were calculated from the resulting changes in OCR (basal and maximal respiration, spare capacity, ATP-linked oxygen consumption, proton leak and non-mitochondrial oxygen consumption). Mean + SD, n=4. *p < 0.05 *vs.* non-treated control by one-way repeated measurement ANOVA corrected by the Holm-Šidák method.

Supplementary Figure 3

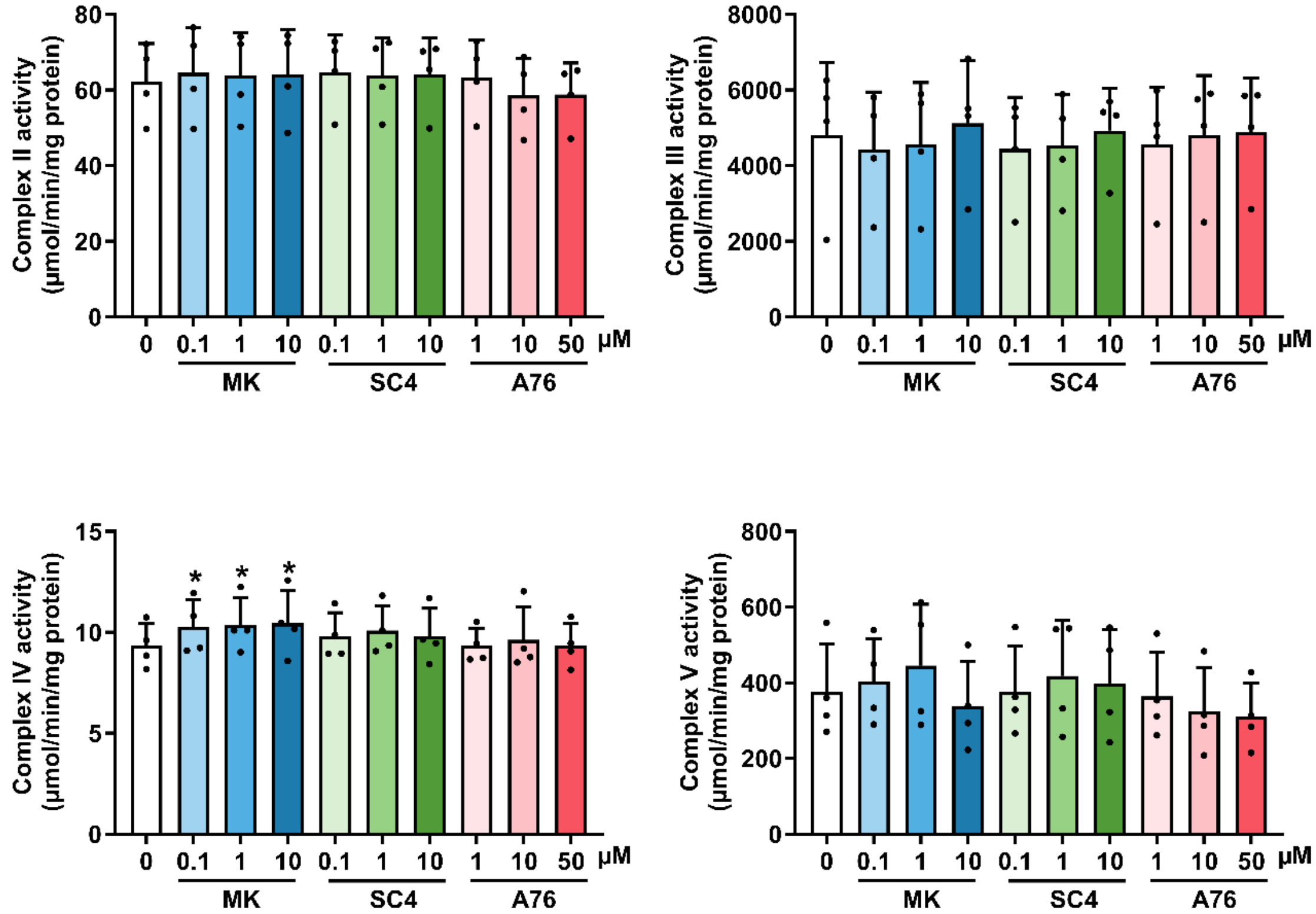

Supplementary Figure 3: Effect of MK-8722, SC4 and A-769662 on respiratory complex activity. Rat heart mitochondria were isolated and subjected to mitochondrial complex activity measurements. MK-8722 (MK), SC4 or A-769662 (A76) were added to the assay at the indicated doses. Individual complex activities were normalised to the protein content of the sample. The activities of complexes II – V are shown. Mean + SD, n=4. *p < 0.05 *vs.* untreated control by one-way repeated measurement ANOVA corrected by the Holm–Šidák method.

Supplementary Figure 4

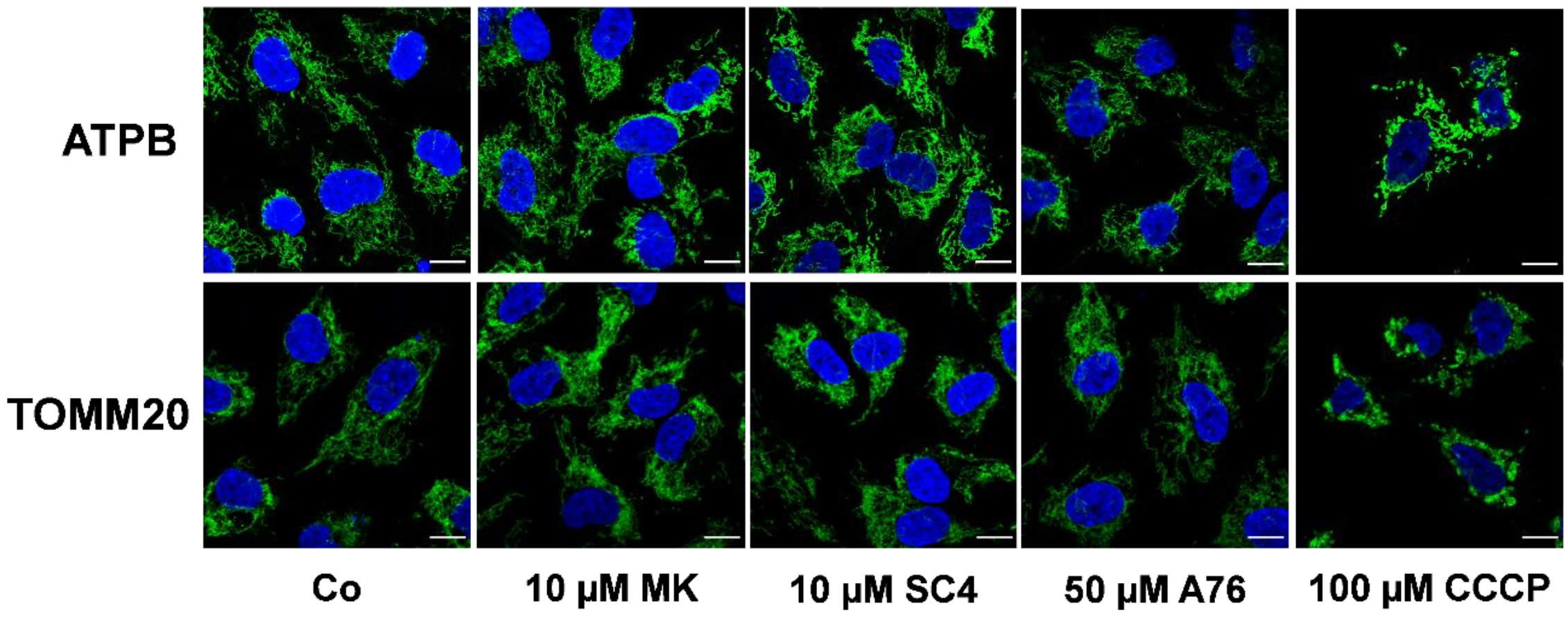

Supplementary Figure 4: Effect of MK-8722, SC4 and A-769662 on morphology. HUVEC were treated with 10 µM MK-8722 (MK), 10 µM SC4 or 50 µM A-769662 (A76) for 24 h. Carbonyl cyanide chlorophenylhydrazone (CCCP, 100 µM, 30 min), was used as a positive control. Mitochondrial morphology was examined by immunofluorescence staining of ATP synthase beta subunit (ATPB, green) (upper part) and of the inner mitochondrial membrane translocase of outer mitochondrial membrane 20 (TOMM20, green) (lower part), a marker of the outer mitochondrial membrane. Nuclei were stained with Hoechst 33342 (blue). Representative images are shown, scale bar = 10 µm. n=3.

Supplementary Figure 5

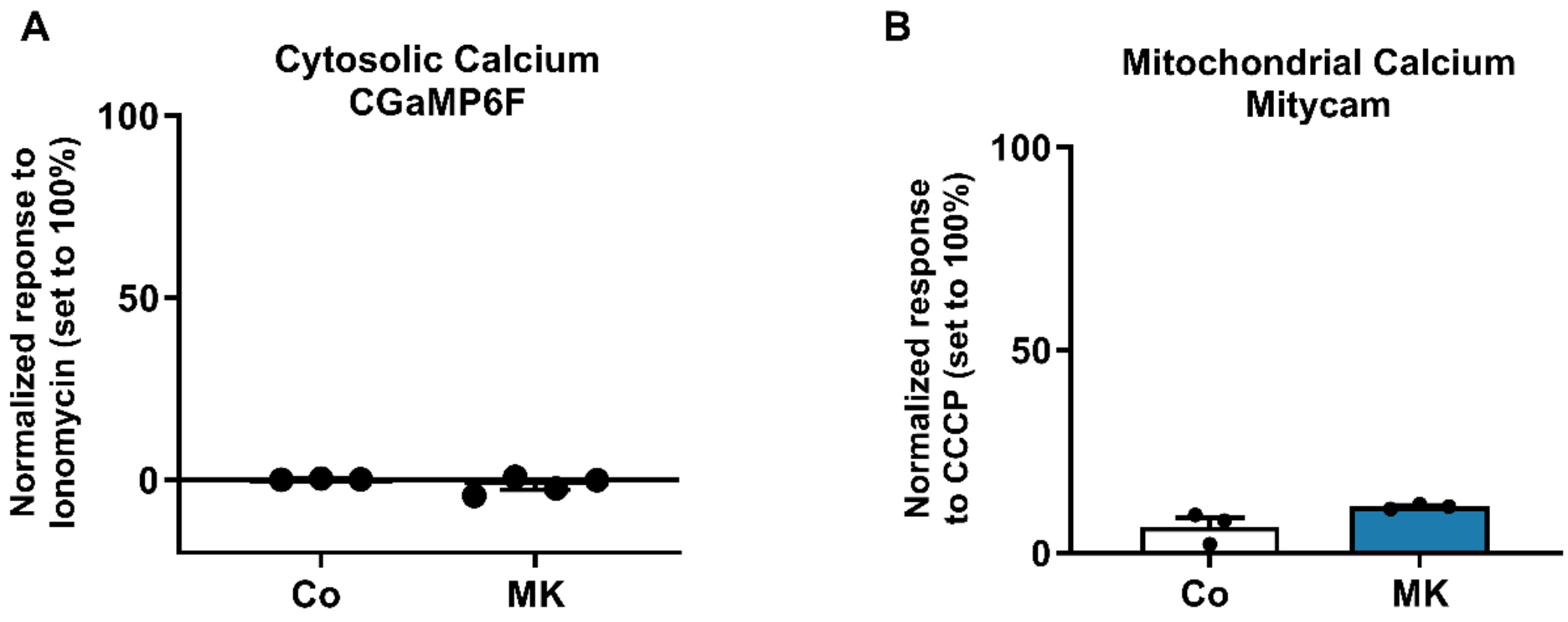

Supplementary Figure 5: Effect of MK-8722 on cytosolic and mitochondrial calcium. HUVEC expressing the cytosolic sensor CGaMP6F (A) or the mitochondrial sensor Mitycam (B) were treated with 10 µM MK-8722 (MK) or DMSO (Co) after starvation for 2 h. Changes in calcium response were recorded using microscopy and normalised to values obtained with ionomycin (1 µM) (A) and carbonyl cyanide chlorophenylhydrazone (CCCP, 100 µM) (B). Mean + SD, n=3-4.

Supplementary Figure 6

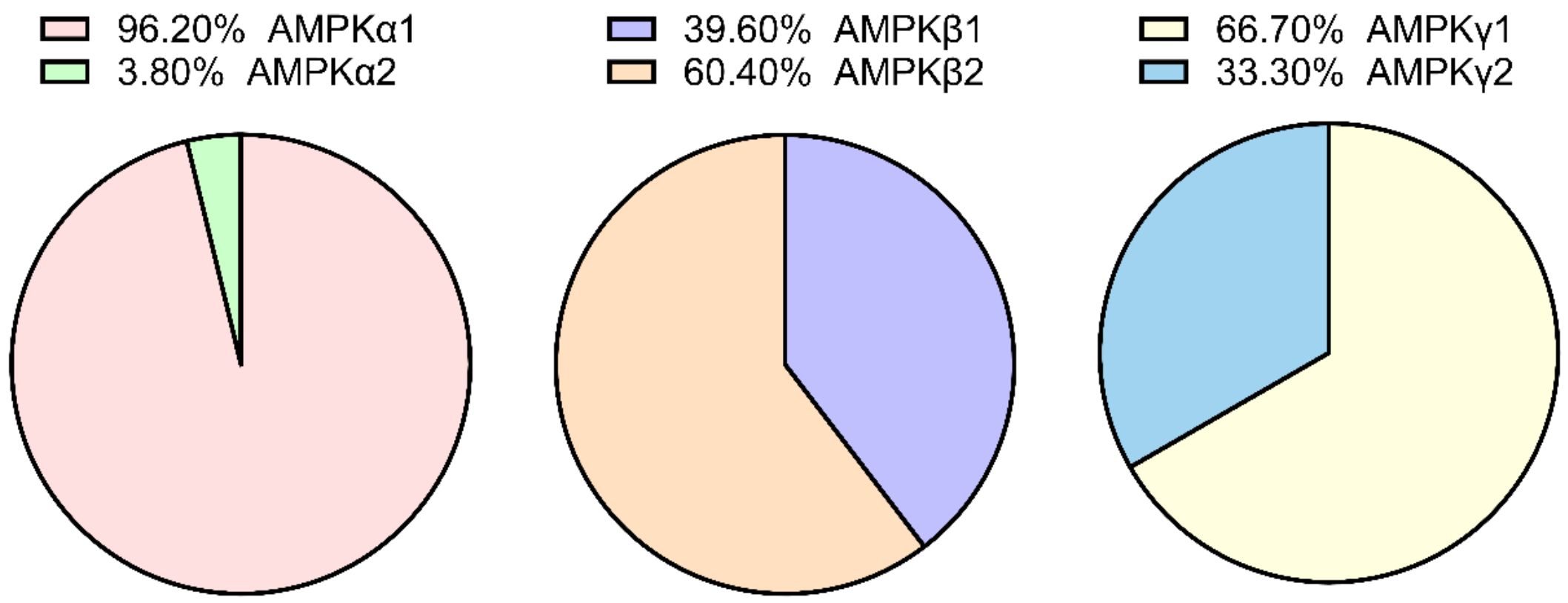

Supplementary Figure 6A: Transcriptomic analysis of AMPK subunit isoform expression in human umbilical vein endothelial cells (HUVEC). The relative expression of AMPK α1/α2, β1/β2, and γ1/γ2/γ3 subunit isoforms was determined from RNA-seq data and calculated as proportional distribution based on reads per kilobase million (RPKM) values. The data are presented as pie charts showing the relative contribution of each isoform within each subunit family. n=4**.** The γ3 subunit was not detectable.

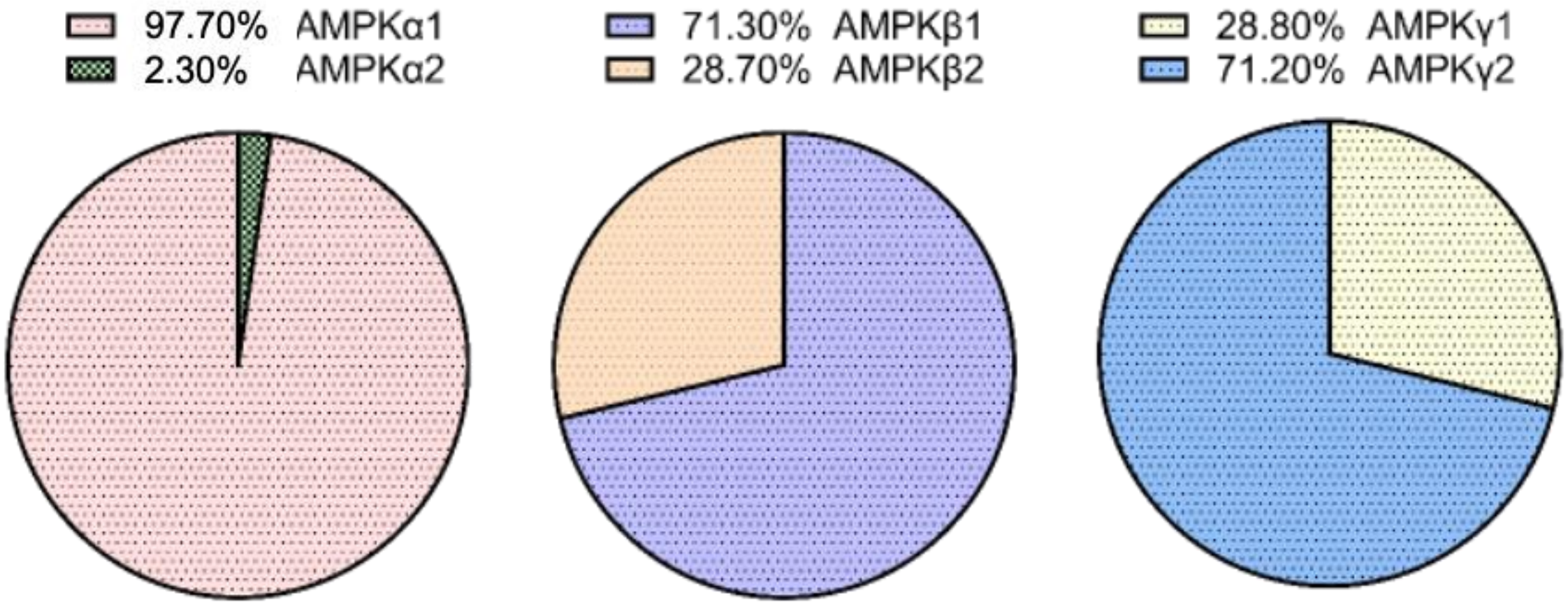

Supplementary Figure 6B: AMPK subunit isoform expression in human umbilical vein endothelial cells (HUVEC) using qRTPCR. RNA was extracted from HUVEC, transcribed into cDNA, and qRT-PCR was performed. The relative expression of AMPK α1/α2, β1/β2, and γ1/γ2/γ3 subunit isoforms normalised to the expression of β-actin is demonstrated, n = 4. The γ3 subunit was not detectable.
